# Culturing marine bacteria from the genus *Pseudoalteromonas* on a cotton scaffold alters secondary metabolite production

**DOI:** 10.1002/mbo3.724

**Published:** 2018-10-01

**Authors:** Marshall L. Timmermans, Katherine J. Picott, Lorena Ucciferri, Avena C. Ross

**Affiliations:** ^1^ Department of Chemistry Queen's University Kingston ON Canada

**Keywords:** biofilm, marine bacteria, metabolite profiling, natural products, proteobacteria, *pseudoalteromonas*

## Abstract

The discovery of secondary metabolites from marine microorganisms is beset by numerous challenges including difficulties cultivating and subsequently eliciting expression of biosynthetic genes from marine microbes in the laboratory. In this paper, we describe a method of culturing three species from the marine bacterial genus *Pseudoalteromonas* using cotton scaffold supplemented liquid media. This simple cultivation method was designed to mimic the natural behavior of some members of the genus wherein they form epibiotic/symbiotic associations with higher organisms such as sponges and corals or attach to solid structures as a biofilm. Our scaffolded cultivation is highly effective at stimulating an attachment/biofilm phenotype and causes large changes to metabolite profiles for the microbes investigated. Metabolite changes include alteration to the production levels of known molecules such as violacein, thiomarinol A, and the alterochromide and prodiginine families of molecules. Finally and critically, our technique stimulates the production of unknown compounds that will serve as leads for future natural product discovery. These results suggest our cultivation approach could potentially be used as a general strategy for the activation of silent gene clusters in marine microbes to facilitate access to their full natural product biosynthetic capacity.

## 1 INTRODUCTION

Mining of microbes for bioactive natural products has resulted in the discovery of a plethora of valuable pharmaceutically relevant compounds (Newman & Cragg, 2016). The incentive to discover or develop novel bioactive molecules from nature has increased significantly in recent years with the rise of antibiotic‐resistant pathogens (Davies & Davies, 2010). Natural product discovery, however, is faced with a number of challenges including activation of silent gene clusters. Natural products discovered through laboratory fermentation of wild‐type strains represent a small fraction of the genetically encoded molecules that exist in nature, as many biosynthetic genes are not expressed at detectable levels under laboratory conditions (Reddy et al., 2012). Furthermore, only a small fraction of microbial strains have been successfully cultivated in the laboratory (Rapp & Giovannoni, 2003; Staley, 1985). Marine microorganisms have received a good deal of attention in recent years as sources of natural product molecules with structural motifs and biosynthetic mechanisms not commonly found among terrestrial bacteria (Montaser & Luesch, 2011; Timmermans, Paudel, & Ross, 2017). However, many marine microorganisms are obligate symbionts with other marine organisms or form biofilms when settled on surfaces. Species adapted to these modes of life can be challenging to culture in vitro*,* and they may differentially express natural product biosynthetic gene clusters under standard laboratory conditions (Berrue, Withers, Haltli, Withers, & Kerr, 2011; Stewart, 2012). It is generally accepted that biofilm formation and quorum sensing are intimately linked for many microbes; however, there are multiple instances where secondary metabolite production is also altered as a result of these behaviors. (Atkinson, Cámara, & Williams, 2007; Barnard et al., 2007; Beauvais & Latgé, 2015; Bleich, Watrous, Dorrestein, Bowers, & Shank, 2015; Braga, Dourado, & Araujo, 2016; Busetti, Maggs, & Gilmore, 2017; Cude & Buchan, 2013; Cude et al., 2015; Favre et al., 2017; Harrington et al., 2014; Johnson, Kido Soule, & Kujawinski, 2016; Nickzad & Déziel, 2014; Othmani, Briand, Ayé, Molmeret, & Culioli, 2016; Zhou, Lyu, Richlen, Anderson, & Caia, 2016). For example, regulation of the biosynthesis of violacein, an antibacterial secondary metabolite produced by several species of *Pseudoalteromonas*, is highly sensitive to acyl‐homoserine lactone (AHL) quorum sensing (Ayé et al., 2015; Wang et al., 2008). We believe that by exploiting the dynamics between biofilm formation, AHL‐mediated quorum sensing, and secondary metabolite production, we can expand laboratory access to microbial bioactive natural products.

**Figure 1:**
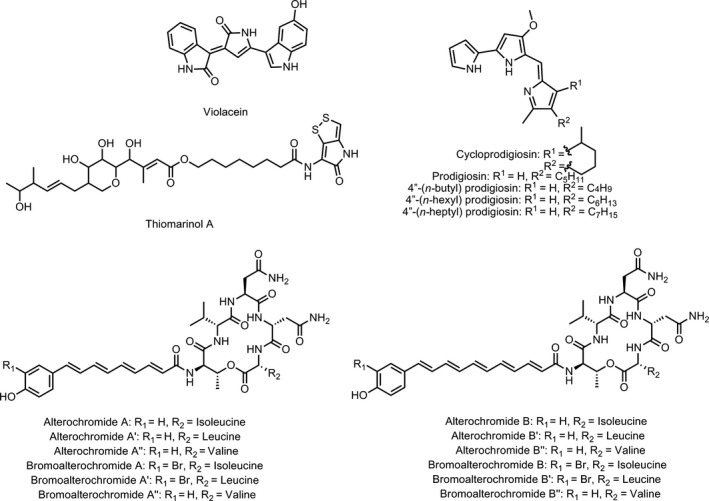
Structures of known secondary metabolites analyzed in this study. Violacein and thiomarinol A, produced by *P. luteoviolacea* 2ta16; the prodiginines, produced by *P. rubra* DSM‐6842; and the alterochromides, produced by *P. piscicida* JCM 20779

Here, we report a set of simple culture conditions that stimulate natural product production by three species of marine gammaproteobacteria of the genus *Pseudoalteromonas* while simultaneously instigating their settlement on a cotton scaffold. *Pseudoalteromonas* is a genus of gram‐negative marine bacteria whose members are found in marine sediment, seawater, and frequently in association with other marine organisms (Skovhus, Holmström, Kjelleberg, & Dahllöf, 2007). Pseudoalteromonads commonly form biofilms for at least part of their life cycle (Rao, Webb, & Kjelleberg, 2005; Sneed, Sharp, Ritchie, & Paul, 2014) and produce a large range of bioactive secondary metabolites including violacein, thiomarinol, pentabromopseudilin, prodigiosin, indolmycin, and bromoalterochromide A (Bowman, 2007; Holmstrom & Kjelleberg, 1999; Vynne, Månsson, Nielsen, & Gram, 2011). In this paper, we detail the effect of cultivation with a cotton scaffold on biofilm formation and the production of the known metabolites violacein, thiomarinol A, the alterochromides, and the prodiginines (Figure 1) by three bacterial species, *Pseudoalteromonas luteoviolacea* 2ta16 (Maansson et al., 2016; Yang, Xiong, Lee, Qi, & Qian, 2007), *P. piscicida* JCM 20779 (Ross, Gulland, Dorrestein, & Moore, 2015; Speitling, Smetanina, Kuznetsova, & Laatsch, 2007), and *P. rubra* DSM‐6842 (Fehér, Barlow, Lorenzo, & Hemscheidt, 2008; Gerber & Gauthier, 1979). We also describe global changes in metabolite profiles for the microbes investigated.

## 2 MATERIALS AND METHODS

### 2.1 Culture conditions

All experiments were performed in biological triplicates, where three separate cultures were each started from a different individual bacterial colony. Cotton ball‐containing cultures were prepared using cotton balls obtained from a national‐chain dollar store (Dollarama, Mont‐Royal, Quebec, Canada). Three balls of cotton wool (~1.5–2.0 g) were selected and placed into acid‐washed 250‐mL Erlenmeyer flasks and sterilized by autoclaving. Post autoclaving, 100 mL of sterile Difco Marine Broth 2216 was aseptically transferred to cotton‐containing flasks.

*Pseudoalteromonas luteoviolacea* 2ta16 and *P. piscicida* JCM 20779 were obtained as a gift from Bradley Moore of the Scripps Institution for Oceanography. These strains were radially streaked onto plates composed of 0.5% (w/v) peptone, 0.3% (w/v) yeast extract, and 2% (w/v) agar in a commercial seawater substitute (Instant Ocean). *P. rubra* DSM‐6842 was obtained from the DSMZ German Collection of Microorganisms and Cell Cultures at the Leibniz Institute. This strain was streaked on agar plates composed of Difco Marine Media 2216 with 2% (w/v) agar. Individual colonies of *P. piscicida* JCM 20779 and *P. luteoviolacea* 2ta16 were picked and used to inoculate separate 5 mL aliquots of liquid media composed of 0.5% (w/v) peptone and 0.3% (w/v) yeast extract, in a commercial artificial seawater substitute (Instant Ocean). Individual colonies of *P. rubra* DSM‐6842 were used to inoculate separate 5 mL aliquots of liquid Difco Marine Media 2216. All liquid cultures were incubated at 30°C for 18 h with shaking at 180 RPM. An aliquot (1 mL) of each overnight culture was then used to inoculate a cotton‐containing culture prepared as described previously, and in parallel a 100 mL culture without cotton balls. All cultures were incubated at 30°C for 18 h with shaking at 180 RPM.

### 2.2 Scanning electron microscopy

Scanning electron microscopy (SEM) was undertaken to observe the formation of biofilms on the surface of cotton fibers. Cotton‐containing cultures of *P. luteoviolacea* 2ta16, *P. piscicida* JCM 20779, and *P. rubra* DSM‐6842 were prepared as described previously. Parallel cultures were allowed to grow for either 24 or 96 h at 30°C with shaking at 180 RPM. Cotton balls were then taken from the cultures and were washed with 50 mL of milli‐Q H_2_O by shaking at 100 RPM in an Erlenmeyer flask for 15 min at 22°C. Cells were fixed by serial washes (50 mL each) with 10% (v/v) ethanol, 25% (v/v) ethanol, 50% (v/v) ethanol, 75% (v/v) ethanol, 90% (v/v) ethanol, and 100% (v/v) ethanol for 15 min each with shaking at 80 RPM at 22°C. Samples were then directly analyzed using an FEI Quanta 250 instrument (operated at 0.6 torr, electron beam operated at 10 kV, Backscatter Electron and Secondary Electron Detectors utilized). SEM images of unused cotton balls were obtained as a control. Unused cotton balls were sputter‐coated with gold and analyzed using the FEI Quanta 250 under identical conditions.

### 2.3 Extraction

After incubation, cultures were divided into three components. An aliquot (50 mL) of the liquid portion of both cotton‐containing and non‐cotton‐containing cultures was centrifuged at 5,000 ***g*** for 5 min. The supernatant of this portion of the culture was extracted with ethyl acetate (3 × 100 mL). The ethyl acetate extract was dried over anhydrous magnesium sulfate and concentrated *in vacuo*. The cell pellet of each culture aliquot was suspended in methanol (50 mL) for 1 h. Insoluble solids were removed by filtration, and the methanol was removed *in vacuo*. Cotton balls were taken out of the culture and excess media removed via vacuum filtration. Cotton balls were then submerged in methanol (50 mL) for 1 h. Insoluble solids were removed by filtration, and methanol was removed *in vacuo*.

### 2.4 Ultraperformance liquid chromatography–photodiode array–mass spectrometry (UPLC‐PDA‐MS) analysis

Crude extracts were dissolved in methanol (1 mL) and analyzed by UPLC‐MS as follows. In the case of *P. luteoviolacea* 2ta16 and *P. rubra* DSM‐6842 extracts, samples were injected onto a Waters UPLC BEH C18 column (1.7 μm particle size, 2.1 × 100 mm) and in the case of *P. piscicida* JCM 20779 extracts were injected onto a Waters UPLC CSH Phenyl‐Hexyl column (1.7 μm particle size, 2.1 × 50 mm). All extracts were separated using a Waters Acquity UPLC system. The UPLC method used a 25 min linear gradient from 65:25:10 H_2_O:MeOH:10% (v/v) formic acid to 0:90:10 H_2_O:MeOH:10% (v/v) formic acid for *P. luteoviolacea* 2ta16 extracts, a 25 minute linear gradient from 80:10:10 H_2_O:MeCN:10% (v/v) formic acid to 0:90:10 H_2_O:MeCN:10% (v/v) formic acid for *P. piscicida* JCM20779 extracts, and a 20 min linear gradient from 70:20:10 H_2_O:MeOH:10% (v/v) formic acid to 0:90:10 H_2_O:MeOH:10% (v/v) formic acid for *P. rubra* DSM‐6842 extracts. Detection was achieved with a photodiode array detector monitoring from 200 to 700 nm and a Waters Acquity single quadrupole mass spectrometer (positive mode scanning between 100 and 2000 *m/z* units in the case of *P. luteoviolacea* 2ta16 extracts, negative ion mode scanning between 250 and 200 *m/z* units in the case of *P. piscicida* JCM 20779 extracts, and positive ion mode scanning between 300 and 450 *m/z* units in the case of *P. rubra* DSM‐6842 extracts.). Mass spectra obtained were then compared to literature values for all known secondary metabolites of the three organisms (Supporting Information Tables S2 and S3) by searching the MarinLit database (Royal Society of Chemistry, 2018). Peak areas of differentially produced compounds were integrated, and comparison was made between biological triplicates of cotton‐containing cultures and biological triplicates of planktonic cultures using a paired Student's *t* test where *α* = 0.05 (Supporting Information Table S4). In the case of the alterochromides, the ratio of peak areas of brominated alterochromides and their non‐brominated analogues was calculated and compared between cotton‐containing cultures and planktonic cultures. Changes in peak area were deemed to be significant when *p* < 0.05.

### 2.5 antiSMASH 4.0 analysis

Genome data from *P. luteoviolacea* 2ta16 (GenBank accession number GCA_000495575.1), *P. piscicida* JCM20779 (GenBank accession number GCA_000238315.4), and *P. rubra* DSM‐6048 (GenBank accession number GCA_000238295.3) were obtained from the National Center for Biotechnology Information (www.ncbi.nlm.nih.gov). Genomes were analyzed using antiSMASH bacterial version 4.0 (Blin et al., 2017). Results are summarized in Supporting Information Table S1.

## 3 RESULTS AND DISCUSSION

To obtain greater access to the encoded secondary metabolites of marine bacteria such as *Pseudoalteromonas luteoviolacea*, we sought to grow bacteria in conditions resembling those in their native habitat by making a simple modification to laboratory culturing conditions. A cotton scaffold in the form of cotton balls was added to the standard liquid culturing conditions to imitate the macroarchitecture of marine invertebrates such as sponges and corals, upon which many microbes reside.

Organic extracts of culture supernatants and biomass were then compared for bacteria grown under standard liquid conditions and the modified cotton scaffold‐liquid conditions. Scanning electron microscopy was used to determine the presence of bacterial biofilms on the cotton scaffold, and ultraperformance liquid chromatography coupled to photodiode array detection with in‐line mass spectrometry (UPLC‐PDA‐MS) was used to determine a metabolite profile for each microbe under each culture condition. Three strains of marine bacteria from the genus *Pseudoalteromonas* were investigated, and the changing production of their known secondary metabolites was documented (Supporting Information Tables S2 and S3).

### 3.1 *Pseudoalteromonas luteoviolacea* 2ta16

**Figure 2:**
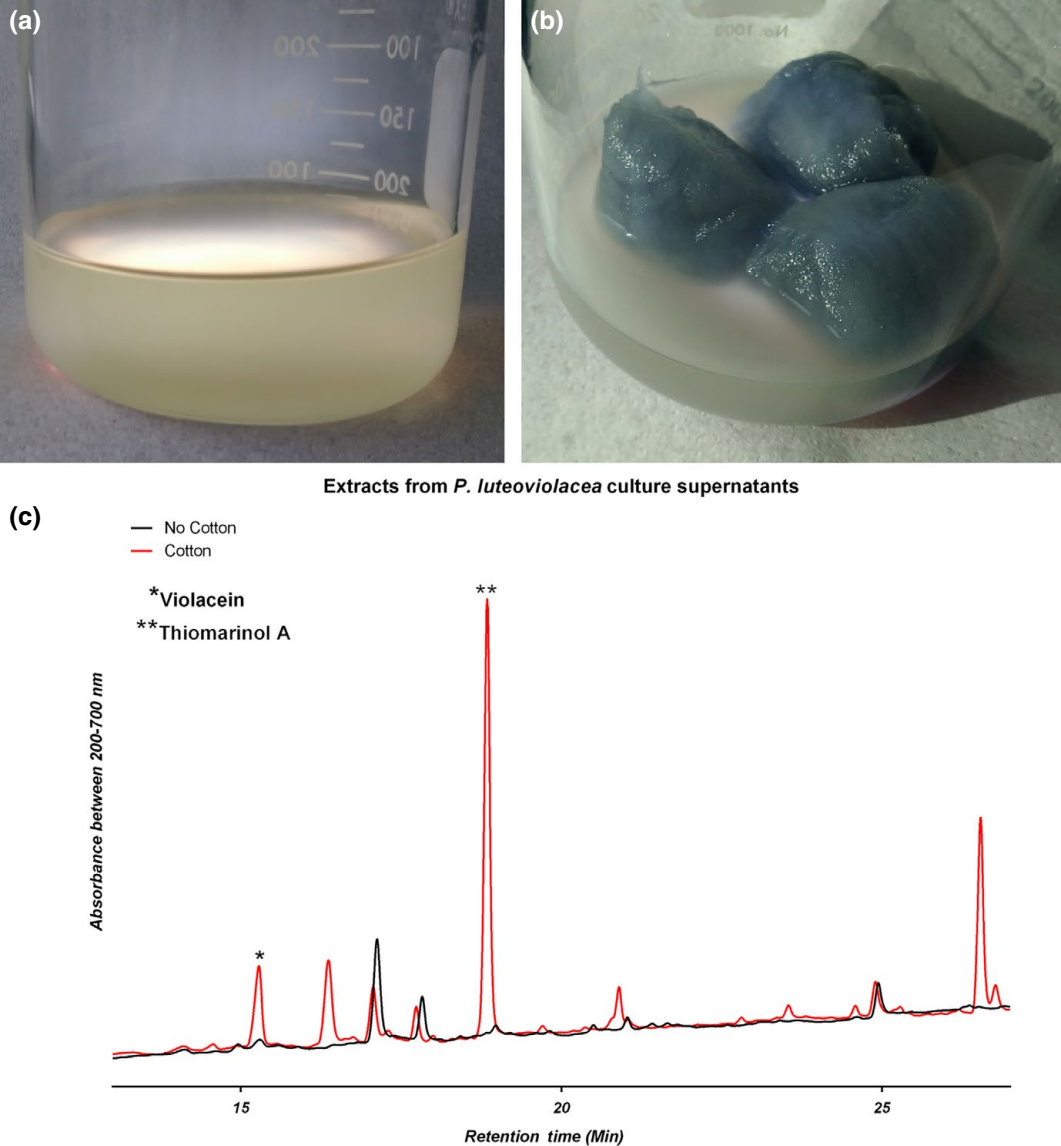
*P. luteoviolacea* 2ta16 grown in Difco Marine Media 2216 in the absence (a) and presence (b) of cotton scaffold. (c): UPLC‐PDA chromatograms of ethyl acetate extracts of cell‐free supernatants of *P. luteoviolacea* 2ta16 cultures showing total UV–Vis absorbance. The peak at 16.1 min has an *m/z* of 344.1 (M+H)^+^ confirming the molecule is violacein. The peak at 19.00 min has an *m/z* of 641.4 (M+H)^+^ confirming the molecule is thiomarinol A

**Figure 3:**
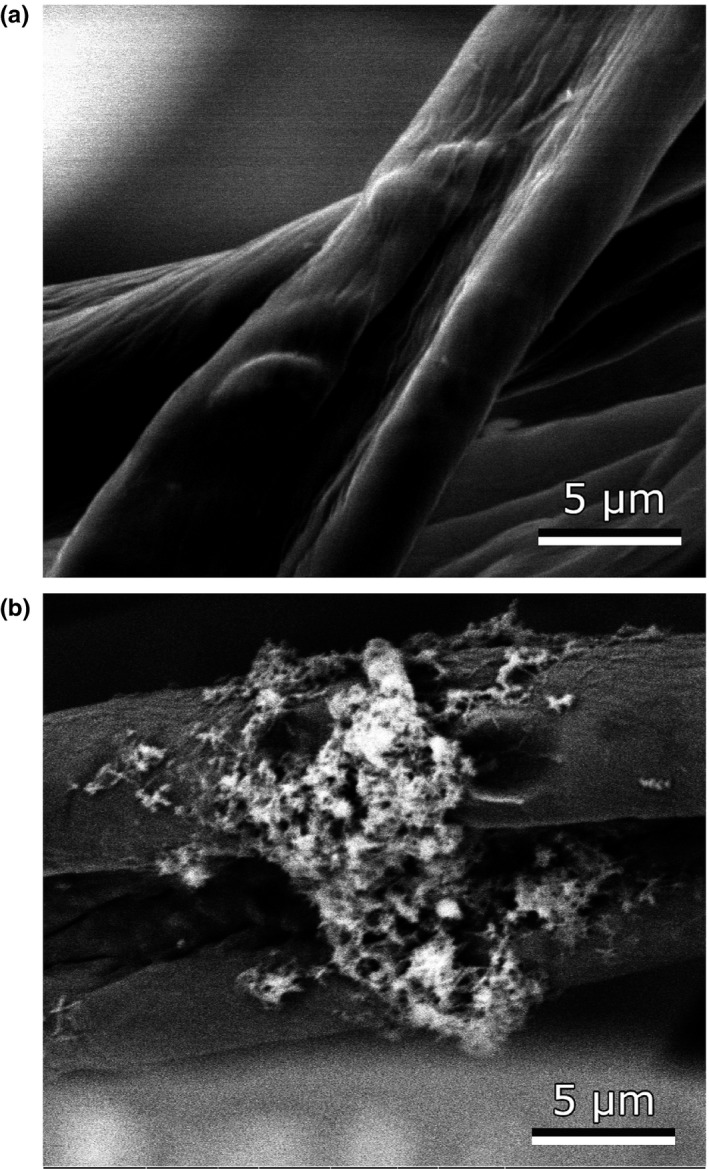
Scanning electron microscope (SEM) images of un‐inoculated cotton balls (a) and cotton balls cultured in the presence of *P. luteoviolacea* 2ta16. In this image, a microcolony of bacterial cells can be seen adhering to the surface of two cotton fibers

*Pseudoalteromonas luteoviolacea* 2ta16 is a γ‐proteobacterial isolate from a coral specimen, *Montastrea anularis,* collected in the Florida Keys (Rypien, Ward, & Azam, 2010). *P. luteoviolacea* 2ta16 is a known producer of several natural products including the bright purple‐coloured antibiotic, violacein (Yang et al., 2007), and the thiomarinol family of dithiolopyrrolone antibiotics (Maansson et al., 2016). Analysis of the *P. luteoviolacea* 2ta16 genome using bioinformatic prediction tool antiSMASH 4.0 (Blin et al., 2017) identified 13 putative biosynthetic gene clusters (Supporting information Table S1), indicating that a number of molecules remain to be discovered from this microbe. In an effort to mimic its native environment, and in the process potentially activate silent natural product biosynthetic gene clusters, we cultivated *P. luteoviolacea* 2ta16 in standard liquid media in the presence and absence of a cotton scaffold. The phenotypic difference between the two cultivations is readily discernible. As seen in Figure 2, when *P. luteoviolacea* is grown in liquid media without cotton, it displays a non‐pigmented phenotype and the bacterial population appears to be entirely planktonic. By contrast, bacteria associated with the cotton in scaffold‐containing cultures demonstrate an intense purple pigmentation and show a mucoid phenotype that is indicative of biofilm formation. SEM images of cotton fibers from this culture show structures consistent with biofilm formation, with clusters of cells forming visible microcolonies on the surface of the fibers (Figure 3 and Supporting information Figure S1). Coverage of cotton fibers was consistent throughout multiple portions of the cotton, with no apparent differences in adherence between the areas of the cotton that were imaged. No obvious morphological differences could be observed between cultures that were grown for 24 h and those grown for 96 h. These images are consistent with the initial phenotypic observations that *P. luteoviolacea* 2ta16 forms a biofilm on the surface of the cotton fibers within 24 hr post‐inoculation.

UPLC‐PDA‐MS analysis of the cell‐free supernatant, planktonic biomass, and cotton‐associated biomass shows distinct differences in the metabolite profiles of *P. luteoviolacea* grown in the two culturing conditions. A peak with mass and UV–Vis profile matching that of violacein appears in significant quantity in the cell‐free supernatant of the cotton scaffold culture (*p*‐value of 0.014) but was undetectable in the supernatant of the non‐cotton‐containing culture as seen in Figure 2. Violacein was also detectable in the planktonic biomass of both cultures but was present in greater quantity in the cotton‐associated biomass as seen in Supporting Information Figure S4. As described in the Introduction, it has previously been reported that violacein biosynthesis in *Pseudoalteromonas* is regulated by quorum sensing molecules including acyl‐homoserine lactones (Wang et al., 2008)*,* and there is an‐established interdependency of quorum sensing and epibiotic biofilm formation in marine bacteria (Zhou et al., 2016). Our SEM images and UPLC‐MS data taken together confirm that the presence of the cotton is facilitating biofilm formation and violacein biosynthesis. These findings support work by Yang and coworkers who observed that cultures of an unidentified strain of *P. luteoviolacea* produced violacein when grown in a stationary manner in which biofilm formation was possible. Contrastingly, cultures grown with agitation, which would inhibit biofilm formation and the associated quorum sensing, did not make violacein. (Yang et al., 2007). The potent dithiolopyrrolone antibiotic thiomarinol A was also readily detected in the cotton‐containing cultures of *P. luteoviolacea* 2ta16 and was undetectable in standard cultures as seen in Figure 2 (*p*‐value 6.0 × 10^−4^, deemed significant) The thiomarinols are produced by a number of *Pseudoalteromonas* strains including *P. luteoviolacea* 2ta16 (Maansson et al., 2016; Murphy et al., 2014; Shiozawa et al., 1993) and although there has been considerable investigation of their biosynthesis (Dunn, Wever, Economou, Bowers, & Li, 2015; Qin, Huang, Yu, & Deng, 2013; Zhai et al., 2016), to the best of our knowledge there have not been reports on the regulation of this biosynthetic pathway or exploration of thiomarinol chemical ecology. Using cotton scaffolds to stimulate molecule production may facilitate examination of these unanswered questions. In our experiments, we also observed that settlement of *P. luteoviolacea* on cotton stimulated the production of a number of other metabolites at levels higher than baseline noise. Initial in‐line UPLC‐MS analysis of these compounds indicates that they are not known secondary metabolites of *P. luteoviolacea* 2ta16. Work is ongoing to isolate and characterize these new natural products. We believe our results culturing *P. luteoviolacea* 2ta16 with a cotton scaffold establish a simple technique for accessing a greater proportion of the encoded biosynthetic potential of marine bacteria. To demonstrate the utility of the approach, we have applied the same cotton scaffold culturing condition to several other *Pseudoalteromonas* species that are not known to be marine invertebrate epibionts.

### 3.2 *Pseudoalteromonas piscicida* JCM20779

*Pseudoalteromonas piscicida* JCM20779 is a γ‐proteobacterium first isolated from a seawater sample collected during a “red tide” event off the west coast of Florida (Bein, 1954). *P*. *piscicida* JCM 20779 is predicted to encode nine biosynthetic gene clusters based on antiSMASH 4.0 analysis (Supporting Information Table S1); however, the only natural products characterized for this strain are the alterochromide family of lipopeptides (Ross et al., 2015). Another strain, *P*. *piscicida* S2040, was recently reported to produce several siderophores (pseudochelin and myxochelins) and the anti‐cancer molecule, alteramide A (Sonnenschein et al., 2017). To assess whether secondary metabolite production by *P*. *piscicida* JCM 20779 is affected by adding a solid support, we grew the strain in liquid media in the presence and absence of a cotton scaffold.

**Figure 4:**
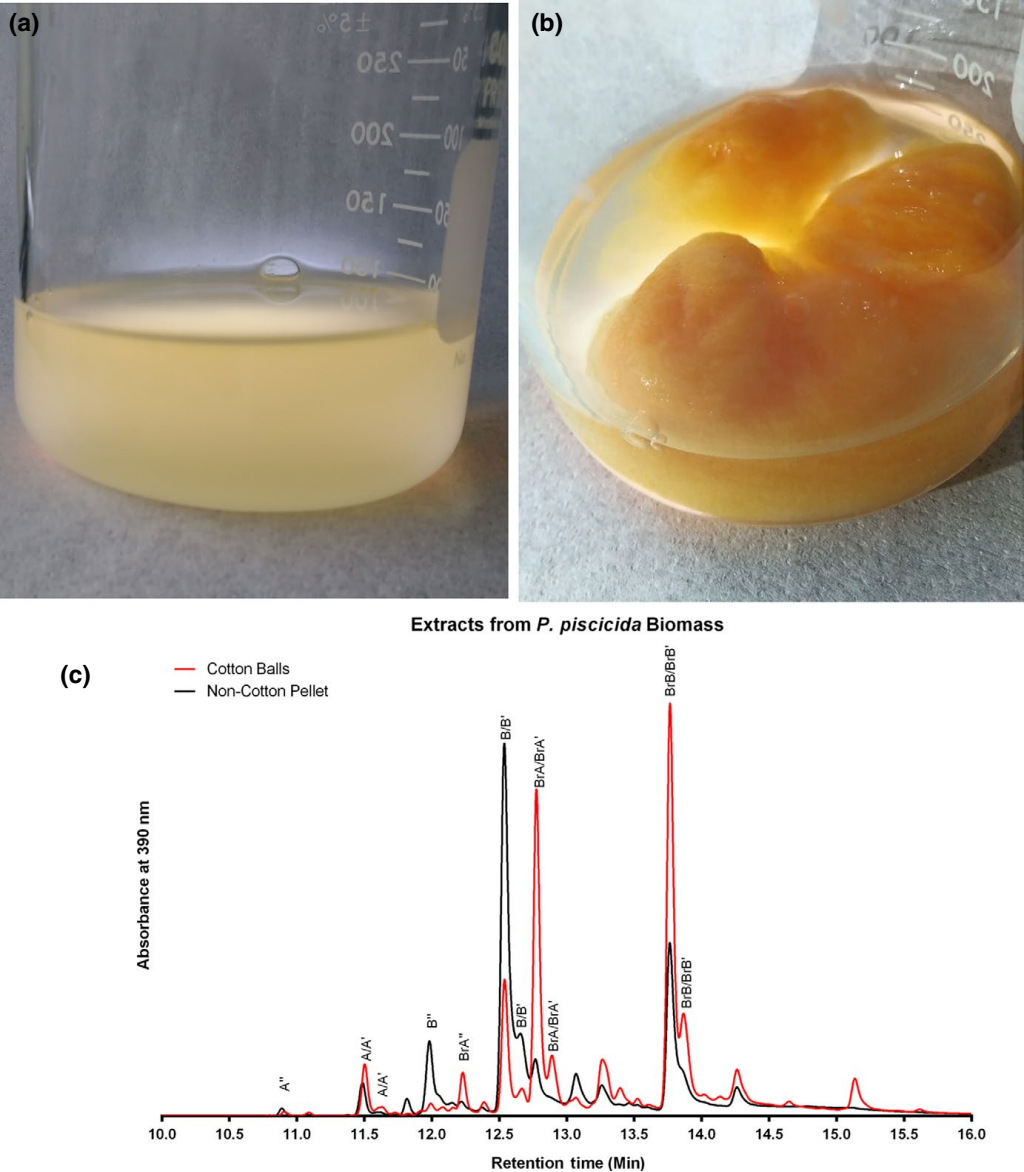
*P. piscicida* JCM 20779 cultured in Difco Marine Media 2216 in the absence (a) and presence (b) of cotton scaffold. (c): Overlaid UPLC‐PDA chromatograms showing absorbance at 390 nm for the methanol extract of the planktonic cell pellet of *P. piscicida* JCM 20779 grown without cotton and the methanol extract of biomass associated with the cotton scaffold for *P. piscicida* JCM 20779 grown with cotton. Known alterochromide variants are indicated. A = alterochromide A, B = alterochromide B, BrA = bromoalterochromide A, BrB = bromoalterochromide B etc

As seen in Figure 4, the addition of cotton balls to liquid culturing conditions results in a phenotypic change to the *P*. *piscicida* JCM 20779. While there is a noticeable orange pigmentation of cells grown in liquid media, growth with added cotton appears to stimulate a considerable increase in pigmentation, especially for cells associated with the cotton. Cells associated with the cotton scaffold also display a mucoid phenotype on air‐exposed surfaces. Based on these observations, it appears that *P. piscicida* JCM 20779 is adhering to the cotton and forming a biofilm. SEM images of *P. piscicida* JCM 20779 can be found in Supporting Information Figure S2 showing a dense coverage of biofilm on cotton fibers from these cultures.

Alterochromide‐like molecules can be catalogued by viewing absorbance chromatograms at 390 nm (Ross et al., 2015). Supporting Information Figure S8 shows UPLC‐PDA chromatograms of ethyl acetate extracts from the cell‐free supernatant, and Supporting Information Figure S9 shows methanol extracts of planktonic cell pellets of cotton‐ and non‐cotton‐containing cultures. These figures show that while it was possible to extract alterochromides from the cell‐free supernatant of the non‐cotton culture, the molecules were not detected in the cell‐free supernatant of the cotton ball cultures. However, alterochromide molecules are clearly detectable in the methanol extracts of biomass from both cotton‐ and non‐cotton‐containing cultures as seen in Figure 4. Indicating that while alterochromides are synthesized in both culture conditions, they do not appear to be secreted from the cells in the cotton treatments.

Although alterochromides are produced under both culture conditions, the relative amounts of different alterochromides differ between cotton and non‐cotton cultures (Figure 4). In‐line mass spectrometry allows for the identification of eight known alterochromide family members. There is a significant increase in the ratios of bromoalterochromide A/A′, bromoalterochromide A″, and bromoalterochromide B/B′ in relation to their non‐brominated analogues when the microbe is cultured with a cotton scaffold (*p* values of 0.024 and 0.028 for the A alterochromides and B alterochromides, respectively). Changes to the alterochromide profile are very consistent between biological replicates.

In addition to the known alterochromides, there are several additional molecules detected only in the cotton ball‐containing cultures that strongly absorb light at 390 nm and show a bromine isotope pattern in their mass spectra. We believe that our new culturing technique allows detection of new alterochromide analogues in addition to changing the relative ratios of known alterochromide variants.

### 3.3 *Pseudoalteromonas rubra* DSM‐6842

*Pseudoalteromonas rubra* DSM‐6842 was originally isolated from a seawater sample from the Mediterranean Sea near Nice, France (Gauthier, 1976). *P. rubra* strains including *P. rubra* DSM‐6842 are known producers of several prodigiosin analogues (Fehér et al., 2008; Gauthier, 1976; Johnson, de Rond, Lindsay, Keasling, & Sarpong, 2015) and analysis of the *P. rubra* DSM‐6842 genome with antiSMASH 4.0 identified 11 putative biosynthetic gene clusters (Supporting Information Table S1). It is important to note that the prodigiosin gene cluster itself is not detected by antiSMASH 4.0, demonstrating a limitation of this prediction tool and emphasizing that the “true” number of encoded secondary metabolites may be underestimated by these types of analyses.

**Figure 5:**
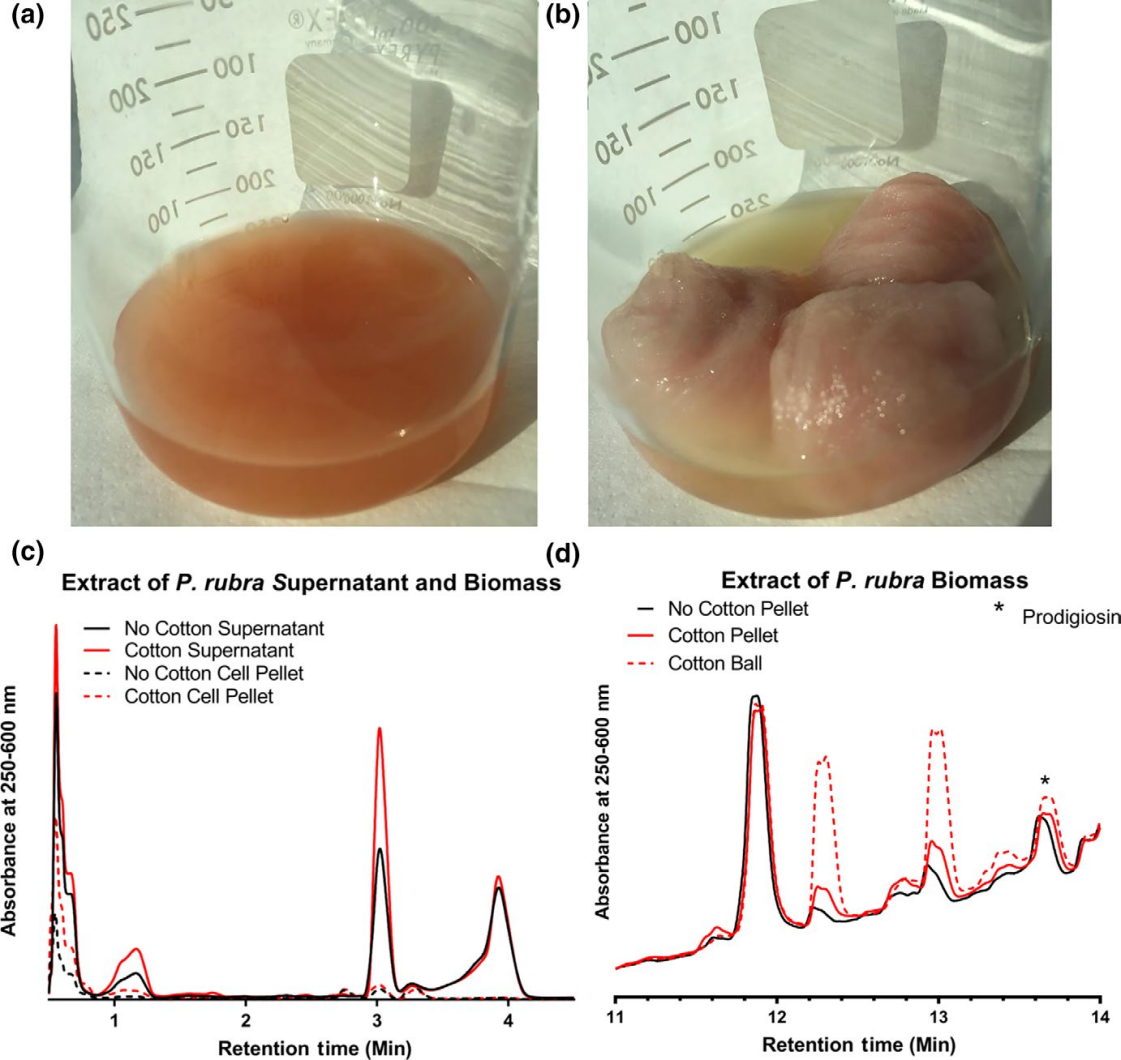
*P. rubra* DSM‐6842 grown in Difco Marine Media 2216 in the absence (a) and presence (b) of cotton scaffold. (c, d): Partial UPLC‐PDA chromatograms showing absorbance at 250–600 nm for organic extracts of *P. rubra* DSM‐6842 grown in the presence and absence of cotton (c) supernatant and biomass extracts, and (d) planktonic and cotton‐associated biomass extracts. The peak at 13.6 min has an *m/z* of 324.1 (M+H)^+^, identifying the compound as prodigiosin

Following our success modifying and increasing secondary metabolite production by Pseudoalteromonads, we cultivated *P. rubra* DSM‐6842 in liquid conditions with and without an added cotton scaffold and analyzed their relative prodiginine production and the global metabolome. Prodiginines are characteristically red in color with an absorbance maximum at 535 nm (Gerber & Gauthier, 1979). Both cultures had visible red pigmentation concentrated in the biomass after centrifugation, clearly indicating prodiginine production and the isolated cotton balls were light pink. Interestingly, the cotton cultures developed a golden yellow coloured supernatant (Figure 5); however, the pigment was not extracted into ethyl acetate, and thus, further experiments will be required to isolate and characterize the corresponding molecule.

In addition to the phenotypic colour differences between cultures, SEM images show the formation of cellular aggregates of *P. rubra* on the surface of cotton fibers (see Supporting Information Figure S3), and changes in the metabolic profiles were also apparent when extracts were analyzed by UPLC‐PDA‐MS. There is a distinct increase in the absorbance at 535 nm for several peaks in the cotton‐associated biomass extract as seen in Supporting Information Figure S13, that were linked to known prodigiosin analogues through mass spectral data (Supporting Information Table S2) (Fehér et al., 2008). The addition of cotton to the culture caused a limited increase in the relative abundance of prodigiosin, 4″‐(*n*‐heptyl) prodigiosin, and cycloprodigiosin production.

The cotton scaffold modified not only production of known prodiginines, but also production of unknown compounds as well. When looking at the global metabolic changes in the supernatant extracts, two peaks at 1.15 min and 3.02 min in the non‐cotton supernatant appeared to increase in intensity in the cotton culture supernatant (Figure 5). Additionally, two initially negligible peaks at 12.27 min and 12.96 min from the planktonic biomass appear to be produced in higher relative quantities in the cotton‐associated biomass (Figure 5). However, it is worth noting that these apparent changes in metabolite production in *P. rubra* were observed in only two out of three biological replicates, demonstrating more variability than was seen for the metabolite profiles of *P. luteoviolacea* 2ta16 or *P. piscicida* JCM 20779 (Supporting Information Figure S4) and affecting statistical analysis (Supporting Information Table S4). The abundance of a bacterial secondary metabolite is a critical factor in the ease of its isolation and identification. With the addition of a cotton scaffold, we display a simple method to upregulate natural product production and therefore facilitate their isolation and characterization.

## 4 CONCLUSIONS

We have found that addition of a cotton scaffold to standard marine bacterial growth media is an effective technique to promote attachment and biofilm formation. This technique may lead to insights into the chemical ecology of the biofilm mode of life in marine bacteria. Cotton settlement appears to be associated with metabolic changes in these Pseudoalteromonads including increases in the proportion of brominated alterochromides produced in relation to their non‐brominated analogues, increased production of thiomarinol A, violacein and potentially prodiginine variants, and the stimulation of synthesis of secondary metabolites not observed for bacteria of the same strain grown in identical media without cotton. It is particularly noteworthy to see secondary metabolite changes observed not only for noted biofilm forming bacterium *P. luteoviolacea* 2ta16*,* but also *P. piscicida* JCM 20779 and *P. rubra* DSM‐6842; organisms that were initially found in their planktonic form and whose biofilms are not described in the literature. Scanning electron microscopy images show that these three strains are forming biofilms on the surface of cotton fibers from our cultures, which is likely the primary driver of the observed changes in secondary metabolite production. However, it is worthy to note that at least two strains of *Pseudoalteromonas* have been isolated that have cellulase activity (Kim et al., 2009; Violot et al., 2005). It is unknown whether the three strains used in this study have any cellulase activity, so further study will be required to determine whether this activity exists and whether it has an effect on secondary metabolite production. Further investigations will focus on elucidating the structure of the unknown metabolites identified in this study and will seek the cause of changes to the metabolite profiles observed here. The question remains whether new metabolites are being observed due to modification to the expression of silent biosynthetic gene clusters or other reasons such as increased cell mass.

Regardless of the mechanism, this method, cotton‐supported culturing, stimulates the production of secondary metabolites not always produced under conventional liquid media growth conditions. This method presents a simple and effective approach for natural product discovery that can likely be applied to many bacterial species.

## CONFLICT OF INTEREST

The authors declare no conflict of interest.

## AUTHOR CONTRIBUTIONS

MLT and ACR designed the experiments; MLT, KJP, and LU carried out the experiments; and MLT and ACR wrote the manuscript with assistance from KJP and LU.

## Data Availability

All data created during this research are openly available from the Scholars Portal Dataverse at https://doi.org/10.5683/sp2/8kb3ue.

## References

[mbo3724-bib-0001] Atkinson, S. , Cámara, M. , & Williams, P. (2007). N‐acylhomoserine lactones, quorum sensing, and biofilm development in gram‐negative bacteria In Kjellberg S. & Givskov M. (Eds.), The biofilm mode of life. Mechanisms and adaptations (pp. 95–122). Wymondham, UK: Horizon Bioscience

[mbo3724-bib-0002] Ayé, M. , Bonnin‐Jusserand, M. , Brian‐Jaisson, F. , Ortalo‐Magné, A. , Culioli, G. , Koffi Nevry, R. , … Molmeret, M. (2015). Modulation of violacein production and phenotypes associated with biofilm by exogenous quorum sensing N‐acylhomoserine lactones in the marine bacterium *Pseudoalteromonas ulvae* TC14. Microbiology, 161, 2039–2052. 10.1099/mic.0.000147 26318530

[mbo3724-bib-0003] Barnard, A. M. L. , Bowden, S. D. , Burr, T. , Coulthurst, S. J. , Monson, R. E. , & Salmond, G. P. (2007). Quorum sensing, virulence and secondary metabolite production in plant soft‐rotting bacteria. Philosophical transactions of the Royal Society of London. Series B, Biological sciences, 362, 1165–1183. 10.1098/rstb.2007.2042 17360277 PMC2435580

[mbo3724-bib-0004] Beauvais, A. , & Latgé, J. (2015). *Aspergillus* biofilm *in vitro* and *in vivo* . Microbiology Spectrum, 3, 1–10. 10.1128/microbiolspec.MB-0017-2015 26350307

[mbo3724-bib-0005] Bein, S. J. (1954). A study of certain chromogenic bacteria isolated from “Red Tide” water with a description of a new species. Bulletin of Marine Science of the Gulf and Caribbean, 4, 110–119.

[mbo3724-bib-0006] Berrue, F. , Withers, S. T. , Haltli, B. , Withers, J. , & Kerr, R. G. (2011). Chemical screening method for the rapid identification of microbial sources of marine invertebrate‐associated metabolites. Marine Drugs, 9, 369–381. 10.3390/md9030369 21556166 PMC3083657

[mbo3724-bib-0007] Bleich, R. , Watrous, J. D. , Dorrestein, P. C. , Bowers, A. A. , Shank, E. A. (2015). Thiopeptide antibiotics stimulate biofilm formation in *Bacillus subtilis* . Proceedings of the National Academy of Sciences, 112, 3086–3091. 10.1073/pnas.1414272112 PMC4364203 25713360

[mbo3724-bib-0008] Blin, K. , Wolf, T. , Chevrette, M. G. , Lu, X. , Schwalen, C. J. , Kautsar, S. A. , … Medema, M. H. (2017). antiSMASH 4. 0 — improvements in chemistry prediction and gene cluster boundary identification. Nucleic Acids Research, 45, 36–41. 10.1093/nar/gkx319 PMC5570095 28460038

[mbo3724-bib-0009] Bowman, J. P. (2007). Bioactive compound synthetic capacity and ecological significance of marine bacterial genus *Pseudoalteromonas* . Marine Drugs, 5, 220–241. 10.3390/md504220 18463726 PMC2365693

[mbo3724-bib-0010] Braga, R. M. , Dourado, M. , Araujo, W. L. (2016). Microbial interactions: Ecology in a molecular perspective. Brazilian Journal of Microbiology, 47, 86–98. 10.1016/j.bjm.2016.10.005 27825606 PMC5156507

[mbo3724-bib-0011] Busetti, A. , Maggs, C. A. , Gilmore, B. F. (2017). Marine macroalgae and their associated microbiomes as a source of antimicrobial chemical diversity. European Journal of Phycology, 52, 452–465. 10.1080/09670262.2017.1376709

[mbo3724-bib-0012] Cude, W. N. , & Buchan, A. (2013). Acyl‐homoserine lactone‐based quorum sensing in the *Roseobacter* clade : complex cell‐to‐cell communication controls multiple physiologies. Frontiers in Microbiology, 4, 1–12. 10.3389/fmicb.2013.00336 24273537 PMC3824088

[mbo3724-bib-0013] Cude, W. N. , Prevatte, C. W. , Hadden, M. K. , May, A. L. , Smith, R. T. , Swain, C. L. , … Buchan, A. (2015). *Phaeobacter* sp. Strain Y4I utilizes two separate cell‐to‐cell communication systems to regulate production of the antimicrobial indigoidine. Applied and Environmental Microbiology, 81, 1417–1425. 10.1128/AEM.02551-14 25527537 PMC4309703

[mbo3724-bib-0014] Davies, J. , & Davies, D. (2010). Origins and evolution of antibiotic resistance. Microbiology and Molecular Biology Reviews, 74, 417–433. 10.1128/MMBR.00016-10 20805405 PMC2937522

[mbo3724-bib-0015] Dunn, Z. D. , Wever, W. J. , Economou, N. J. , Bowers, A. A. , & Li, B. (2015). Enzymatic basis of “Hybridity” in thiomarinol biosynthesis. Angewandte Chemie (International ed. in English), 54, 5137–5141. 10.1002/anie.201411667 25726835 PMC4462198

[mbo3724-bib-0016] Favre, L. , Ortalo‐Magné, A. , Greff, S. , Pérez, T. , Thomas, O. P. , Martin, J. C. , & Culioli, G. (2017). Discrimination of four marine biofilm‐forming bacteria by LC‐MS metabolomics and influence of culture parameters. Journal of Proteome Research, 16, 1962–1975. 10.1021/acs.jproteome.6b01027 28362105

[mbo3724-bib-0017] Fehér, D. , Barlow, R. S. , Lorenzo, P. S. , & Hemscheidt, T. K. (2008). A 2‐substituted prodiginine, 2‐(p‐Hydroxybenzyl) prodigiosin, from *Pseudoalteromonas rubra* . Journal of Natural Products, 71, 1970–1972. 10.1021/np800493p 18922034 PMC2891405

[mbo3724-bib-0018] Gauthier, M. J. (1976). *Alteromonas rubra* sp. nov., a new marine antibiotic‐producing bacterium. International Journal of Systematic Bacteriology, 26, 459–466. 10.1099/00207713-26-4-459

[mbo3724-bib-0019] Gerber, N. N. , & Gauthier, M. J. (1979). New prodigiosin‐like pigment from *Alteromonas rubra* . Applied and Environment Microbiology, 37, 1176–1179. 10.1128/aem.37.6.1176-1179.1979 PMC243374 384909

[mbo3724-bib-0020] Harrington, C. , Jerry Reen, F. , Mooij, M. J. , Stewart, F. A. , Chabot, J. B. , Guerra, A. F. , … O'Gara, F. (2014). Characterisation of Non‐Autoinducing Tropodithietic Acid (TDA) production from marine sponge *Pseudovibrio* species. Marine Drugs, 12, 5960–5978. 10.3390/md12125960 25513851 PMC4278212

[mbo3724-bib-0021] Holmstrom, C. , & Kjelleberg, S. (1999). Marine *Pseudoalteromonas* species are associated with higher organisms and produce biologically active extracellular agents. FEMS Microbiology Ecology, 30, 285–293. 10.1016/S0168-6496(99)00063-X 10568837

[mbo3724-bib-0022] Johnson, R. E. , de Rond, T. , Lindsay, V. N. , Keasling, J. D. , & Sarpong, R. (2015). Synthesis of cycloprodigiosin identifies the natural isolate as a scalemic mixture. Organic Letters, 17, 3474–3477. 10.1021/acs.orglett.5b01527 26114660 PMC4509414

[mbo3724-bib-0023] Johnson, W. M. , Kido Soule, M. C. , & Kujawinski, E. B. (2016). Evidence for quorum sensing and differential metabolite production by a marine bacterium in response to DMSP. ISME Journal, 10, 2304–2316. 10.1038/ismej.2016.6 26882264 PMC4989321

[mbo3724-bib-0024] Kim, D. , Baik, K. S. , Park, S. C. , Kim, S. J. , Shin, T. S. , Jung, S. J. , … Seong, C. N. (2009). Cellulase production from *Pseudoalteromonas* sp. NO_3_ isolated from the sea squirt *Halocynthia rorentzi* . Journal of Industrial Microbiology and Biotechnology, 36, 1375–1382. 10.1007/s10295-009-0623-y 19639353

[mbo3724-bib-0025] Maansson, M. , Vynne, N. G. , Klitgaard, A. , Nybo, J. L. , Melchiorsen, J. , Nguyen, D. D. , … Gram, L. (2016). An integrated metabolomic and genomic mining workflow to uncover the biosynthetic potential of bacteria. mSystems, 1, e00028–15. 10.1128/mSystems.00028-15 PMC5069768 27822535

[mbo3724-bib-0026] Montaser, R. , & Luesch, H. (2011). Marine natural products: A new wave of drugs? Future Medicinal Chemistry, 3, 1475–1489. 10.4155/fmc.11.118 21882941 PMC3210699

[mbo3724-bib-0027] Murphy, A. C. , Gao, S. S. , Han, L.‐C. , Carobene, S. , Fukuda, D. , Song, Z. , … Simpson, T. J. (2014). Biosynthesis of thiomarinol A and related metabolites of *Pseudoalteromonas* sp. SANK 73390. Chemical Science, 5, 397–402. 10.1039/C3SC52281D

[mbo3724-bib-0028] Newman, D. J. , & Cragg, G. M. (2016). Natural products as sources of new drugs from 1981 to 2014. Journal of Natural Products, 79, 629–661. 10.1021/acs.jnatprod.5b01055 26852623

[mbo3724-bib-0029] Nickzad, A. , & Déziel, E. (2014). The involvement of rhamnolipids in microbial cell adhesion and biofilm development – an approach for control? Letters in Applied Microbiology, 58, 447–453. 10.1111/lam.12211 24372465

[mbo3724-bib-0030] Othmani, A. , Briand, J. F. , Ayé, M. , Molmeret, M. , Culioli, G. (2016). Surface metabolites of the brown alga *Taonia atomaria* have the ability to regulate epibiosis. Biofouling, 32, 801–813. 10.1080/08927014.2016.1198954 27353006

[mbo3724-bib-0031] Qin, Z. , Huang, S. , Yu, Y. , & Deng, H. (2013). Dithiolopyrrolone natural products: Isolation, synthesis and biosynthesis. Marine Drugs, 11, 3970–3997. 10.3390/md11103970 24141227 PMC3826145

[mbo3724-bib-0032] Rao, D. , Webb, J. S. , & Kjelleberg, S. (2005). Competitive interactions in mixed‐species biofilms containing the marine bacterium *Pseudoalteromonas tunicata* . Applied and Environment Microbiology, 71, 1729–1736. 10.1128/AEM.71.4.1729-1736.2005 PMC1082554 15811995

[mbo3724-bib-0033] Rapp, M. S. , & Giovannoni, S. J. (2003). The uncultured microbial majority. Annual Review of Microbiology, 57, 369–394. 10.1146/annurev.micro.57.030502.090759 14527284

[mbo3724-bib-0034] Reddy, B. V. , Kallifidas, D. , Kim, J. H. , Charlop‐Powers, Z. , Feng, Z. , & Brady, S. F. (2012). Natural product biosynthetic gene diversity in geographically distinct soil microbiomes. Applied and Environment Microbiology, 78, 3744–3752. 10.1128/AEM.00102-12 PMC3346347 22427492

[mbo3724-bib-0035] Ross, A. C. , Gulland, L. E. , Dorrestein, P. C. , & Moore, B. S. (2015). Targeted capture and heterologous expression of the *Pseudoalteromonas* alterochromide gene cluster in *Escherichia coli* represents a promising natural product exploratory platform. ACS Synthetic Biology, 4, 414–420. 10.1021/sb500280q 25140825 PMC4410906

[mbo3724-bib-0036] Royal Society of Chemistry (2018). MarinLit – A database of the marine natural products literature. [Online]. Retrieved from http://pubs.rsc.org/marinlit/. [Accessed: 15‐May‐2018]

[mbo3724-bib-0037] Rypien, K. L. , Ward, J. R. , & Azam, F. (2010). Antagonistic interactions among coral‐associated bacteria. Environmental Microbiology, 12, 28–39. 10.1111/j.1462-2920.2009.02027.x 19691500

[mbo3724-bib-0038] Shiozawa, H. , Kagasaki, T. , Kinoshita, T. , Haruyama, H. , Domon, H. , Utsui, Y. , … Takahashi, S. (1993). Thiomarinol, a new hybrid antimicrobial antibiotic produced by a marine bacterium: Fermentation, isolation, structure, and antimicrobial activity. Journal of Antibiotics (Tokyo), 46, 1834–1842. 10.7164/antibiotics.46.1834 8294241

[mbo3724-bib-0039] Skovhus, T. L. , Holmström, C. , Kjelleberg, S. , & Dahllöf, I. (2007). Molecular investigation of the distribution, abundance and diversity of the genus *Pseudoalteromonas* in marine samples. FEMS Microbiology Ecology, 61, 348–361. 10.1111/j.1574-6941.2007.00339.x 17573938

[mbo3724-bib-0040] Sneed, J. M. , Sharp, K. H. , Ritchie, K. B. , & Paul, V. J. (2014). The chemical cue tetrabromopyrrole from a biofilm bacterium induces settlement of multiple Caribbean corals. Proceedings of the Royal Society B, 281, 20133086 10.1098/rspb.2013.3086 24850918 PMC4046396

[mbo3724-bib-0041] Sonnenschein, E. C. , Stierhof, M. , Goralczyk, S. , Vabre, F. M. , Pellissier, L. , Hanssen, K. Ø. , … Tabudravu, J. N. (2017). Pseudochelin A, a siderophore of *Pseudoalteromonas piscicida* S2040. Tetrahedron, 73, 2633–2637. 10.1016/j.tet.2017.03.051

[mbo3724-bib-0042] Speitling, M. , Smetanina, O. F. , Kuznetsova, T. A. , & Laatsch, H. (2007). Bromoalterochromides A and A’, unprecedented chromopeptides from a marine *Pseudoalteromonas maricaloris* Strain KMM 636. Journal of Antibiotics (Tokyo), 60, 36–42. 10.1038/ja.2007.5 17390587

[mbo3724-bib-0043] Staley, J. T. (1985). Measurement of *in situ* activities of nonphotosynthetic microorganisms in aquatic and terrestrial habitats. Annual Review of Microbiology, 39, 321–346. 10.1146/annurev.mi.39.100185.001541 3904603

[mbo3724-bib-0044] Stewart, E. J. (2012). Growing unculturable bacteria. Journal of Bacteriology, 194, 4151–4160. 10.1128/JB.00345-12 22661685 PMC3416243

[mbo3724-bib-0045] Timmermans, M. L. , Paudel, Y. P. , & Ross, A. C. (2017). Investigating the biosynthesis of natural products from marine proteobacteria: A survey of molecules and strategies. Marine Drugs, 15, E235 10.3390/md15080235 28762997 PMC5577590

[mbo3724-bib-0046] Violot, S. , Aghajari, N. , Czjzek, M. , Feller, G. , Sonan, G. K. , Gouet, P. , … Receveur‐Bréchot, V. (2005). Structure of a full length psychrophilic cellulase from *Pseudoalteromonas haloplanktis* revealed by X‐ray diffraction and small angle X‐ray scattering. Journal of Molecular Biology, 348, 1211–1224. 10.1016/j.jmb.2005.03.026 15854656

[mbo3724-bib-0047] Vynne, N. G. , Månsson, M. , Nielsen, K. F. , & Gram, L. (2011). Bioactivity, chemical profiling, and 16S rRNA‐based phylogeny of *Pseudoalteromonas* strains collected on a global research cruise. Marine Biotechnology, 13, 1062–1073. 10.1007/s10126-011-9369-4 21305330

[mbo3724-bib-0048] Wang, Y. , Ikawa, A. , Okaue, S. , Taniguchi, S. , Osaka, I. , Yoshimoto, A. , … Enomoto, K. (2008). Quorum sensing signaling molecules involved in the production of violacein by *Pseudoalteromonas* . Bioscience, Biotechnology, and Biochemistry, 72, 1958–1961. 10.1271/bbb.80090 18603795

[mbo3724-bib-0049] Yang, L. H. , Xiong, H. , Lee, O. O. , Qi, S. H. , & Qian, P. Y. (2007). Effect of agitation on violacein production in *Pseudoalteromonas luteoviolacea* isolated from a marine sponge. Letters in Applied Microbiology, 44, 625–630. 10.1111/j.1472-765X.2007.02125.x 17576224

[mbo3724-bib-0050] Zhai, Y. , Bai, S. , Liu, J. , Yang, L. , Han, L. , Huang, X. , & He, J. (2016). Identification of an unusual type II thioesterase in the dithiolopyrrolone antibiotics biosynthetic pathway. Biochemical and Biophysical Research Communications, 473, 329–335. 10.1016/j.bbrc.2016.03.105 27018252

[mbo3724-bib-0051] Zhou, J. , Lyu, Y. , Richlen, M. , Anderson, D. M. , & Caia, Z. (2016). Quorum Sensing is a language of chemical signals and plays an ecological role in algal‐bacterial interactions. CRC. Critical Reviews in Plant Sciences, 35, 81–105. 10.1080/07352689.2016.1172461 28966438 PMC5619252

