# Supplementary material for: Culturing marine bacteria from the genus Pseudoalteromonas on a cotton scaffold alters secondary metabolite production
Source: Microbiologyopen. 2018 Oct 1;8(5):e00724. doi: 10.1002/mbo3.724 (PMC6528606; doi:10.1002/mbo3.724)
Supplement: Supplementary file 1 [file MBO3-8-e00724-s001.pdf]

Supplementary Information for "Culturing marine bacteria from the genus Pseudoalteromonas on a cotton scaffold activates silent biosynthetic pathways and alters secondary metabolite production"

Marshall L. Timmermans, Katherine J. Picott, Lorena Ucciferri, Avena C. Ross

AntiSmash 4.0 Analysis of Bacterial Genomes

Table S1. Type of secondary metabolite biosynthetic gene clusters detected in *Pseudoalteromonas* sp. genome sequence data by antiSMASH 4.0 analysis [1].

Type of natural product pathway	*P. luteoviolacea* 2ta16 (137 scaffolds)	*P. piscicida* JCM 20779 (2 scaffolds)	*P. rubra* DSM 6842 (194 scaffolds)*
RiPPs	2	3	2
Non-ribosomal peptide (NRP)	3	2	3
Polyketide (PK)			1
Hybrid (NRP/PK)	4	4 (including alterochromide pathway)	1
Lantipeptide	1		2
Homo serine lactone	1		1
Indole	1 (violacein pathway)		
Aryl polyene cluster	1		
Other			1

* Prodiginine pathway from *P. rubra* was not detected through antiSMASH 4.0

Scanning Electron Microscopy

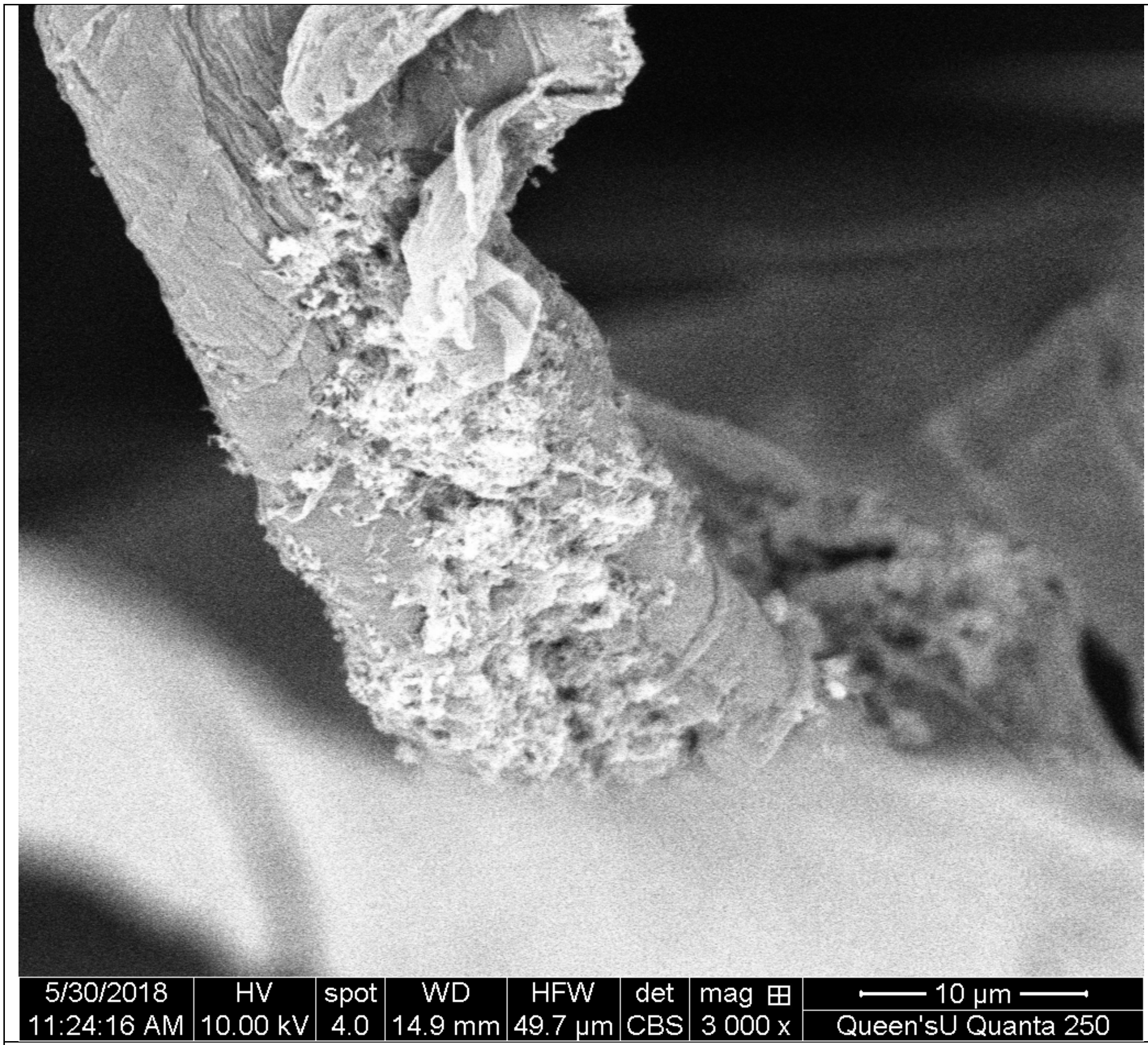

Figure S1 Scanning Electron Micrograph of cotton fibres with *P. luteoviolacea* 2ta16 biofilm adhering to its surface.

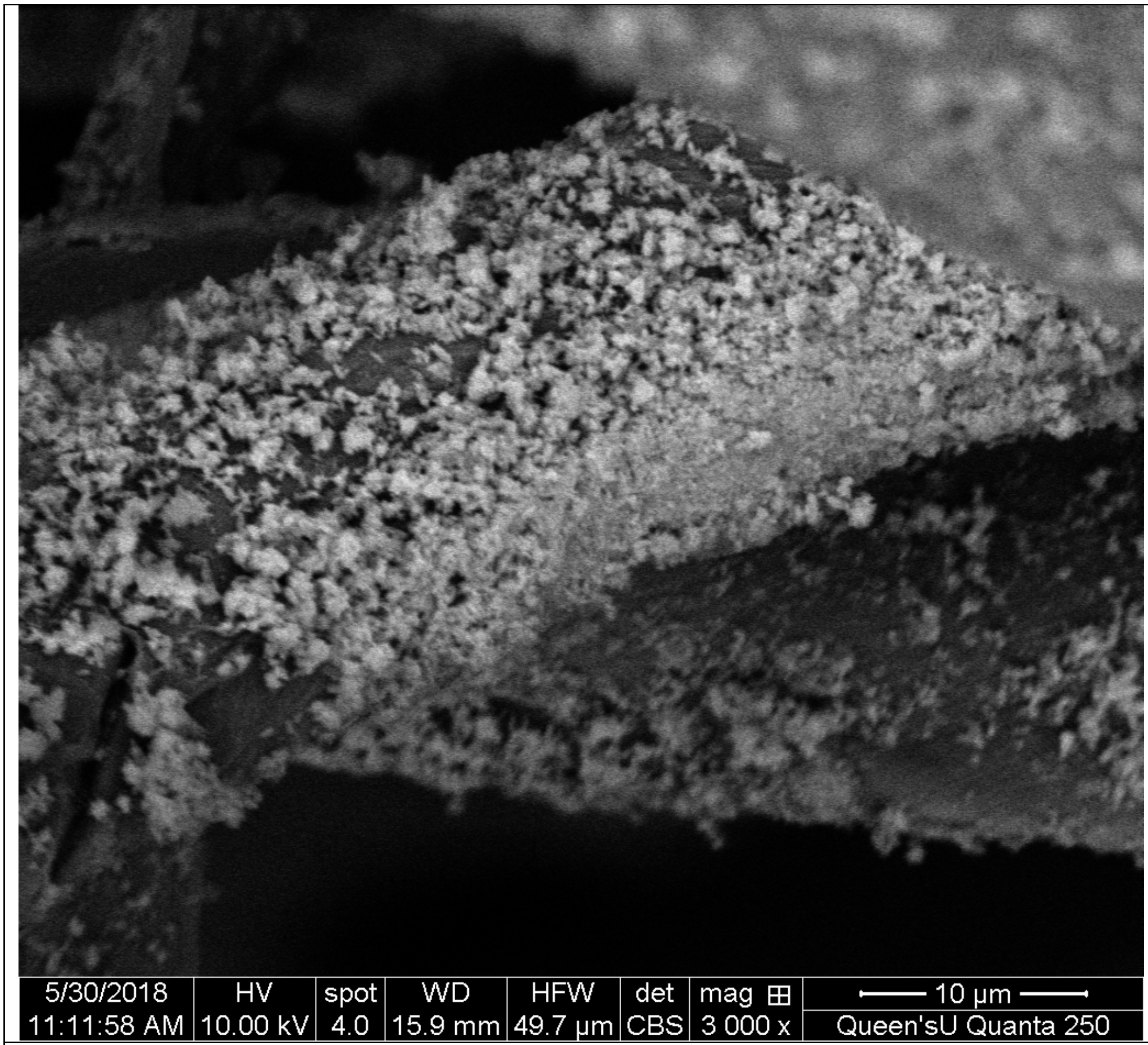

Figure S2: Scanning Electron Micrograph of cotton fibres with *P. piscicida* JCM 20779 biofilm adhering to its surface.

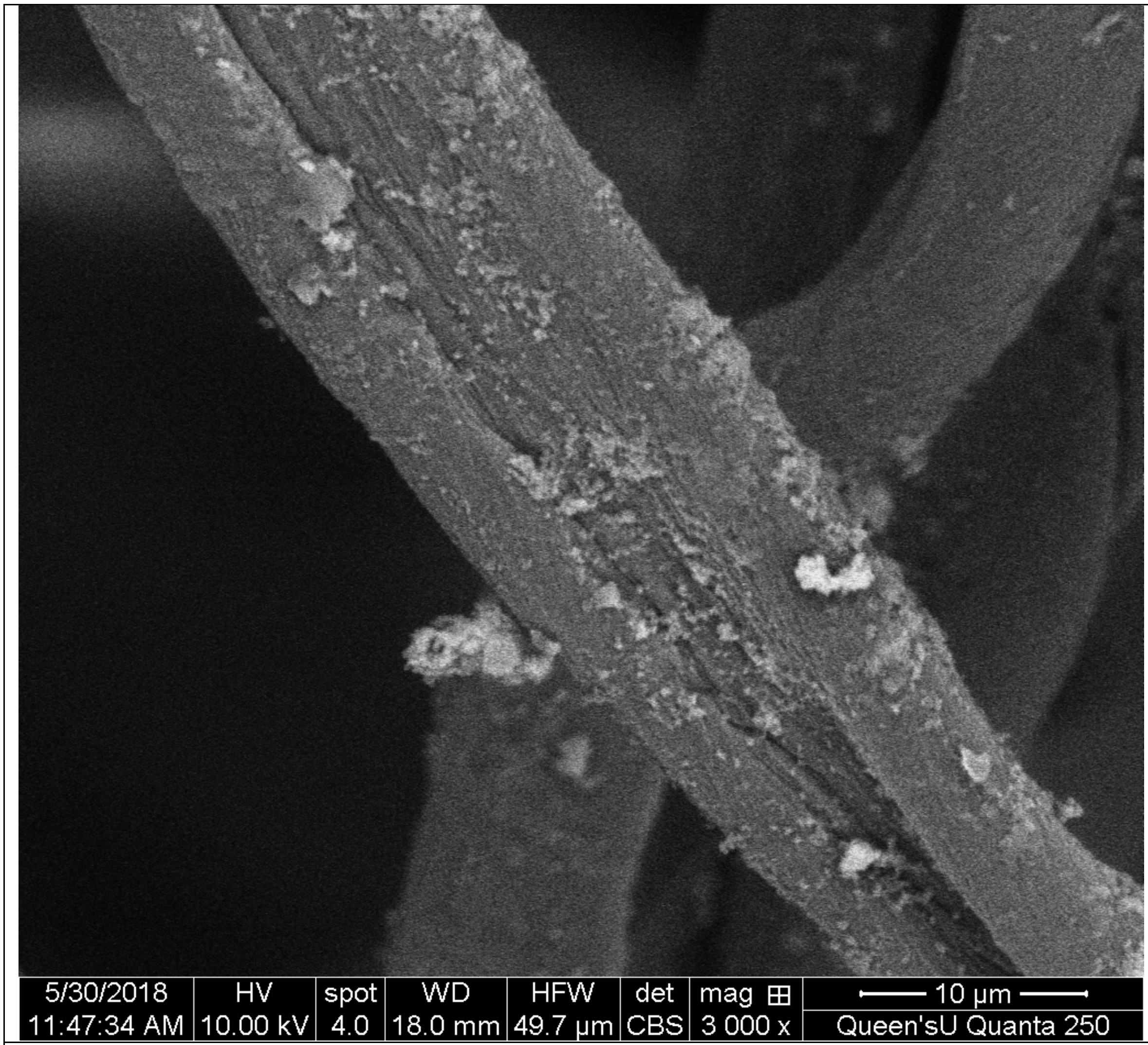

Figure S3: Scanning Electron Micrograph of cotton fibres with *P. rubra* DSM-6842 biofilm adhering to its surface. Microcolonies are visible as large clusters of cells, individual cells are also seen adhering to the surface of the cotton fibres.

***P. luteoviolacea* 2ta16**

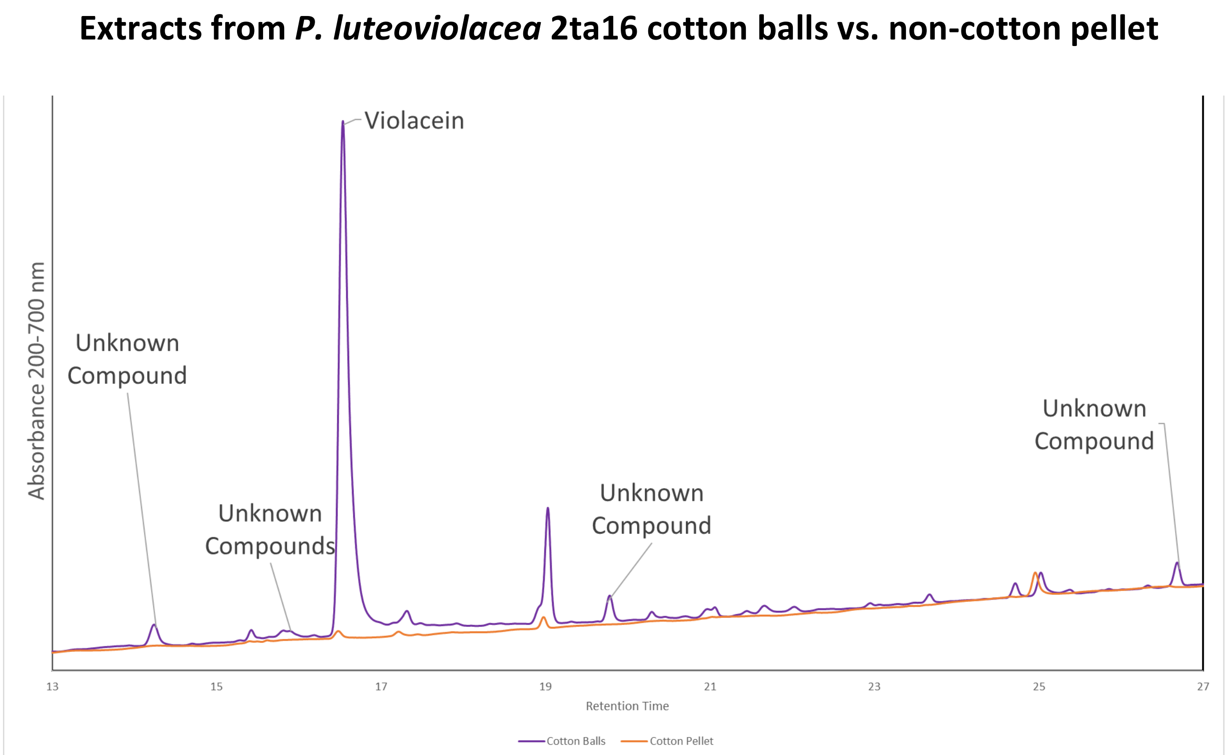

Figure S4: UPLC-PDA trace of methanol extracts of cotton balls from cotton containing culture, as well as methanol extract of cell pellets from non-cotton containing cultures of *P. luteoviolacea* 2ta16.

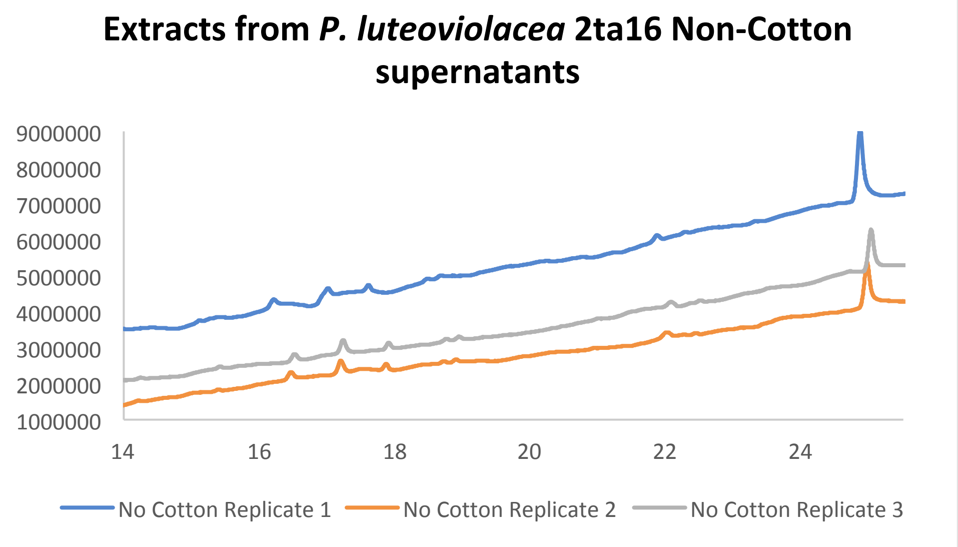

Figure S5: UPLC-PDA Trace of ethyl-acetate extracts from biological replicates of planktonic cultures of *P. luteoviolacea* 2ta16.

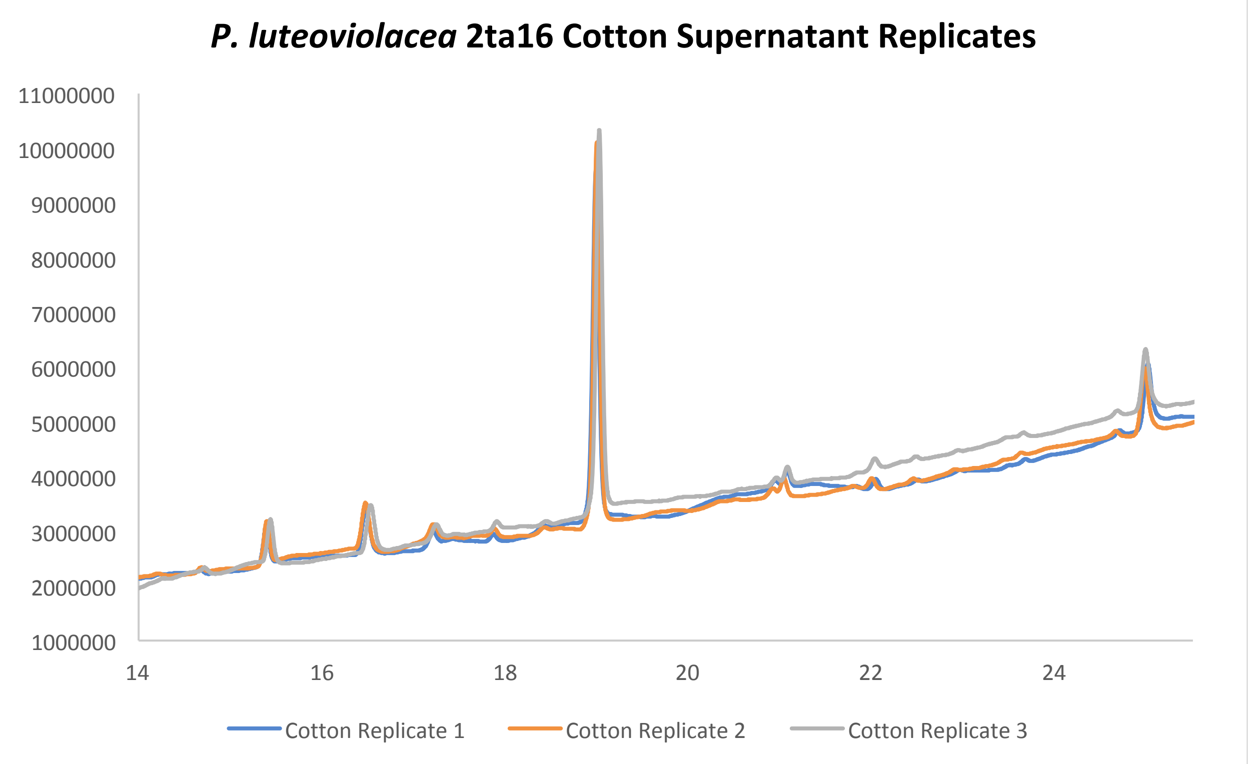

Figure S6: UPLC-PDA Trace of ethyl-acetate extracts from biological replicates of cotton containing cultures of *P. luteoviolacea* 2ta16.

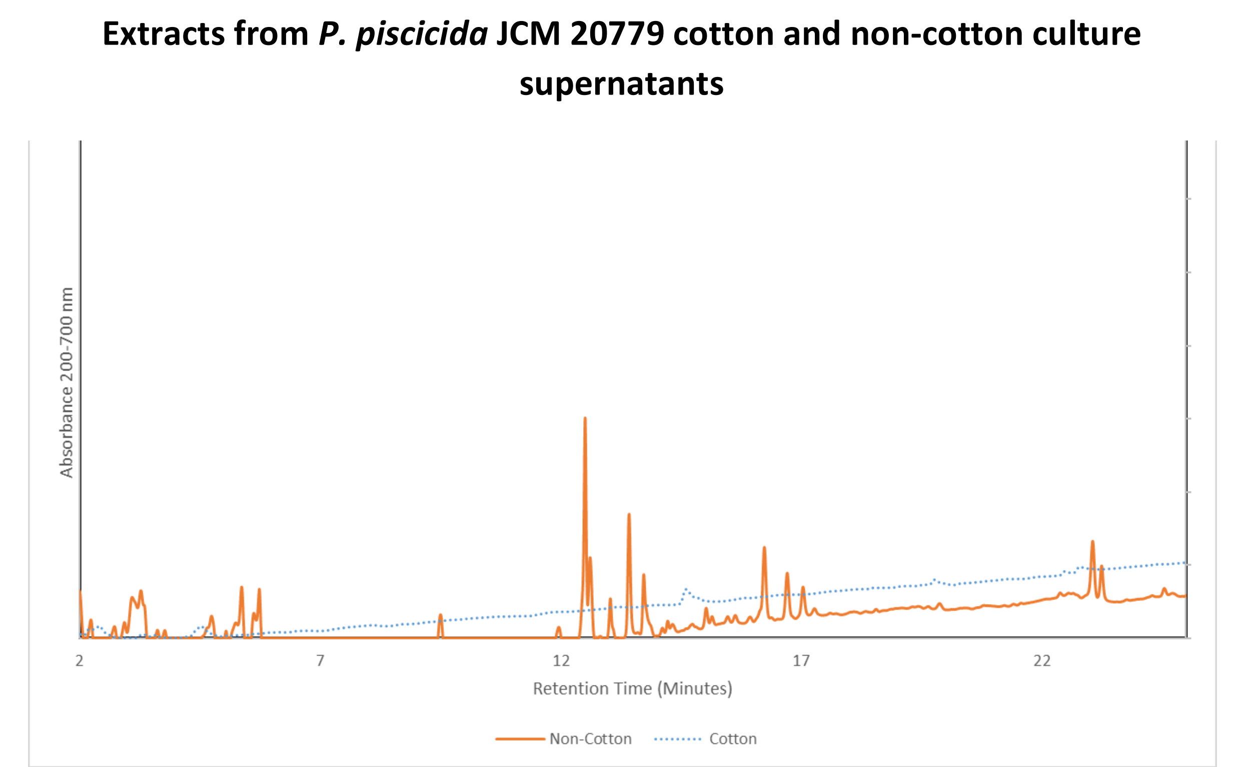

Figure S7: UPLC-PDA trace of ethyl acetate extracts of cell free supernatants from *P.piscicida* JCM 20779

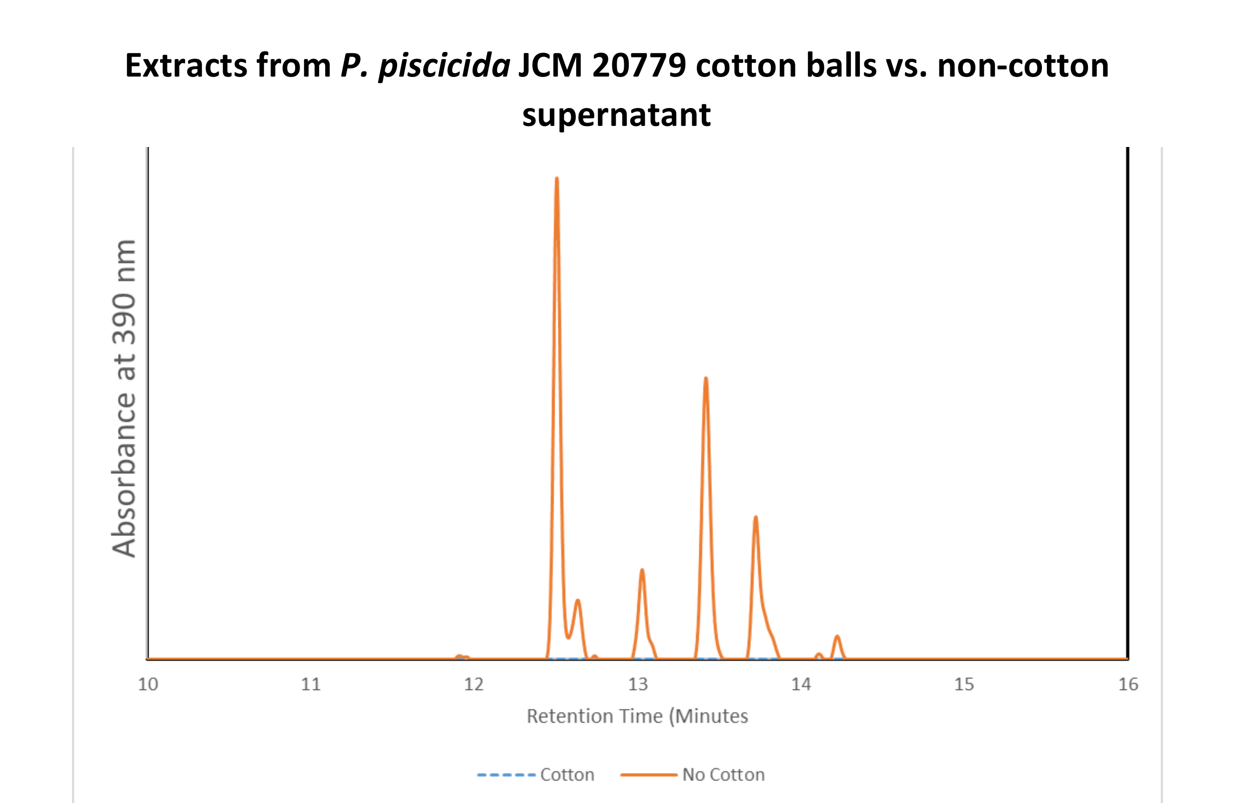

Figure S8: UPLC-PDA chromatograms of ethyl acetate extracts of cell-free supernatants from cultures of *P. piscicida* JCM 20779 grown with and without cotton. Absorbance at 390 nm is indicative of alterochromide family members.

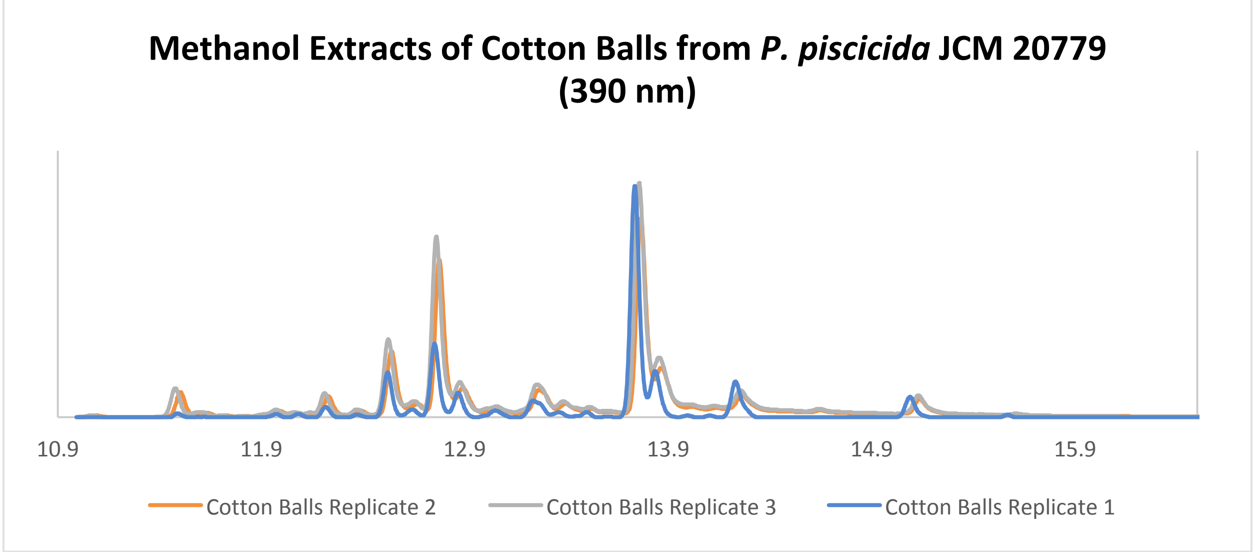

Figure S9: UPLC-PDA Trace of methanol extracts from biological replicates of cotton containing cultures of *P. piscicida* JCM 20779.

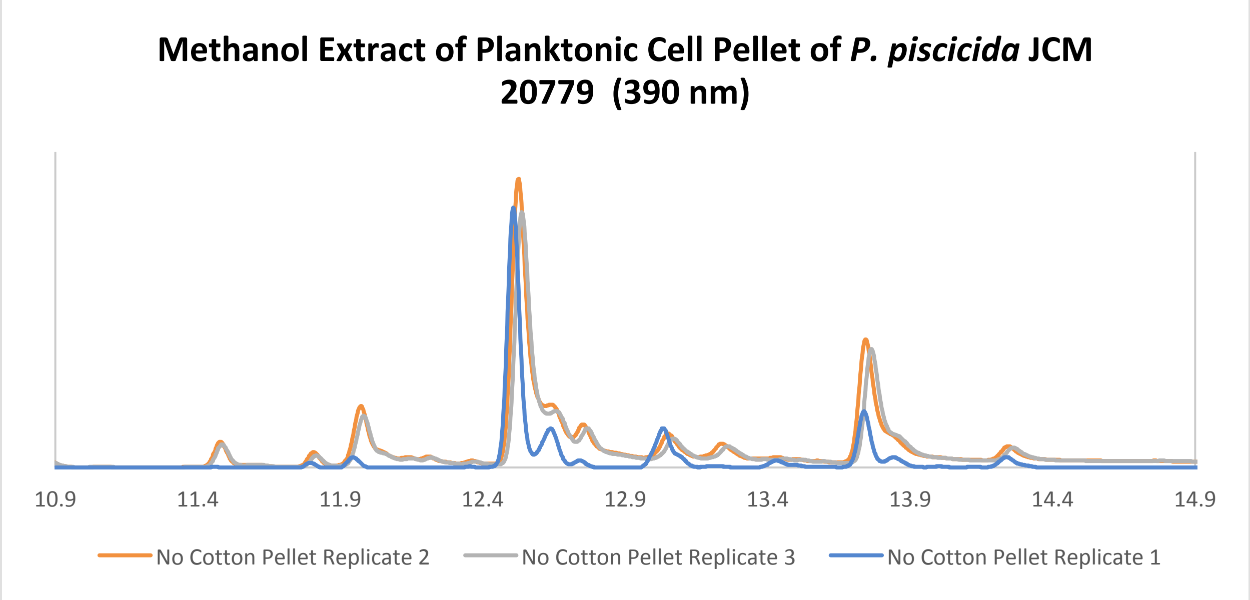

Figure S10: UPLC-PDA Trace of methanol extracts from biological replicates of planktonic cultures of *P. piscicida* JCM 20779.

***P.rubra* DSM-6842**

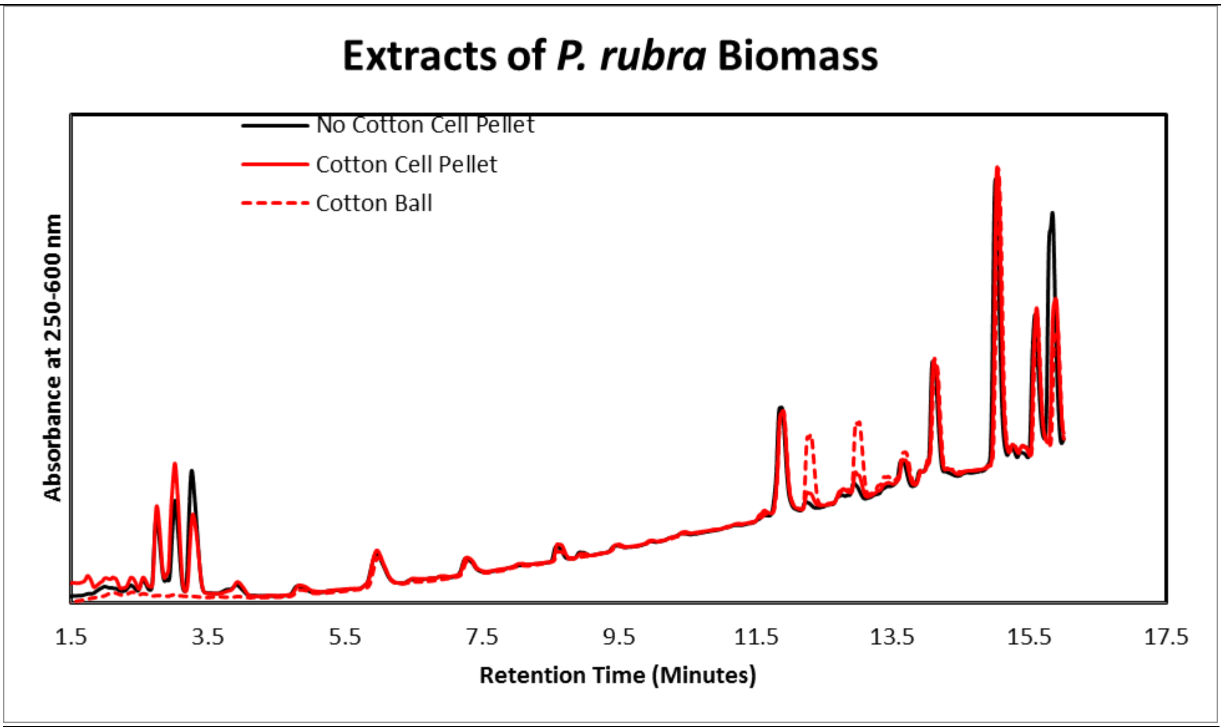

Figure S11: Averaged UPLC-PDA chromatograms of methanol extracts of *P. rubra* DSM-6842 biomass from cultures containing cotton scaffold and cultures lacking cotton scaffold.

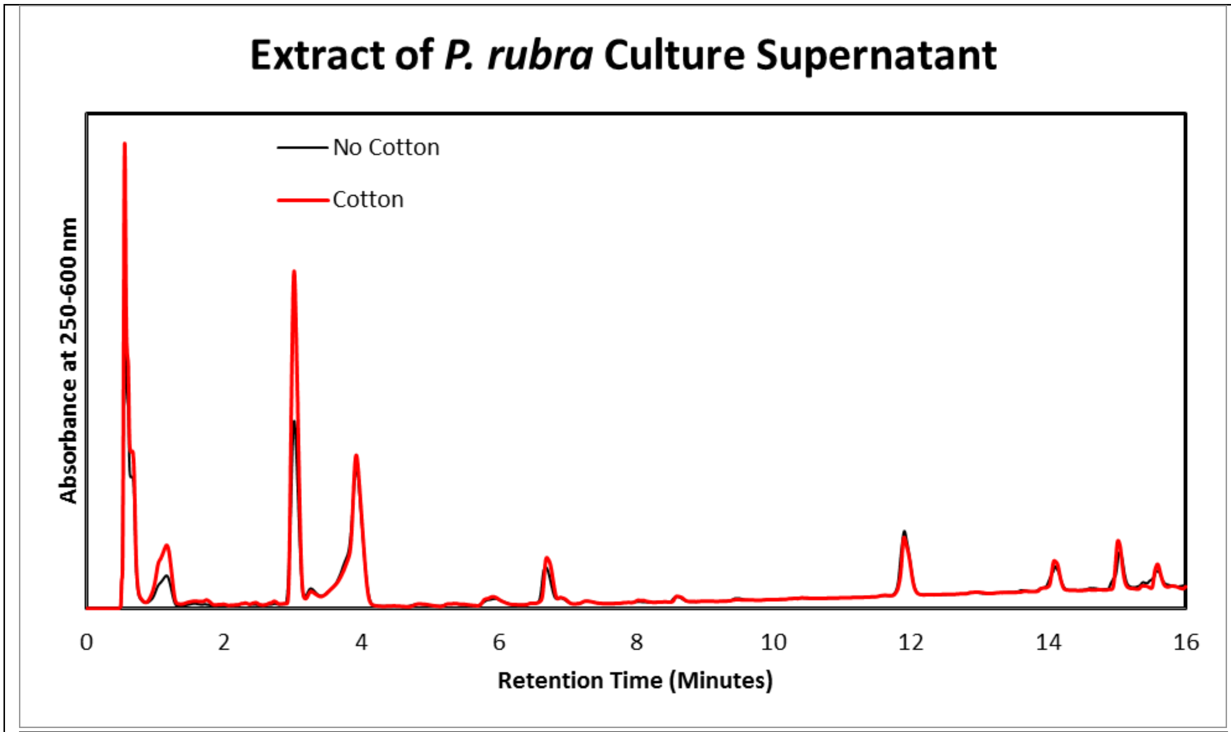

Figure S12: Averaged UPLC-PDA chromatograms of ethyl acetate extracts of cell-free supernatants from cultures of *P. rubra* DSM-6842 grown with and without cotton scaffolds.

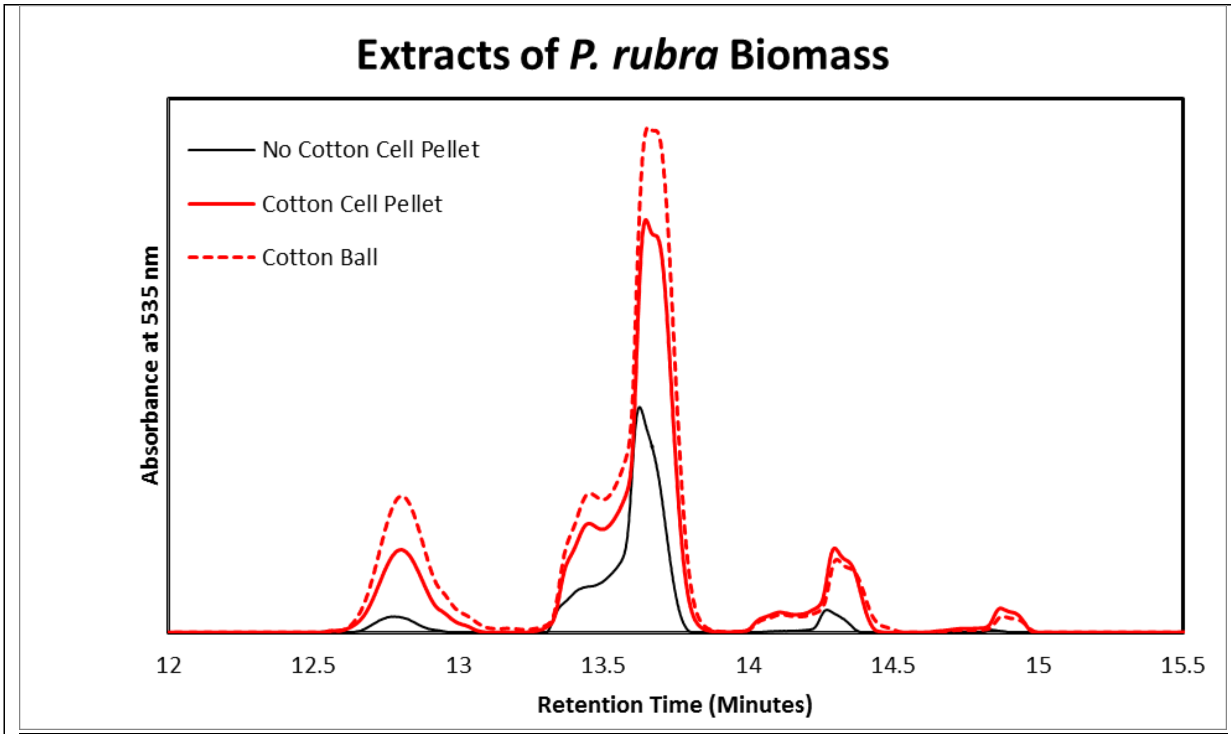

Figure S13: Averaged UPLC-PDA chromatograms of extracts from the biomass of cultures of *P. rubra* DSM-6842 grown with and without cotton scaffolds. Compounds identified are Cycloprodigiosin/4’’-(n-butyl)prodigiosin (Co-eluted at 12.8 minutes, Prodigiosin (RT = 13.69 minutes), and 4’’-(n-hexyl)prodigiosin (RT = 14.35), and 4’’-(n-heptyl)prodigiosin (RT = 14.87)

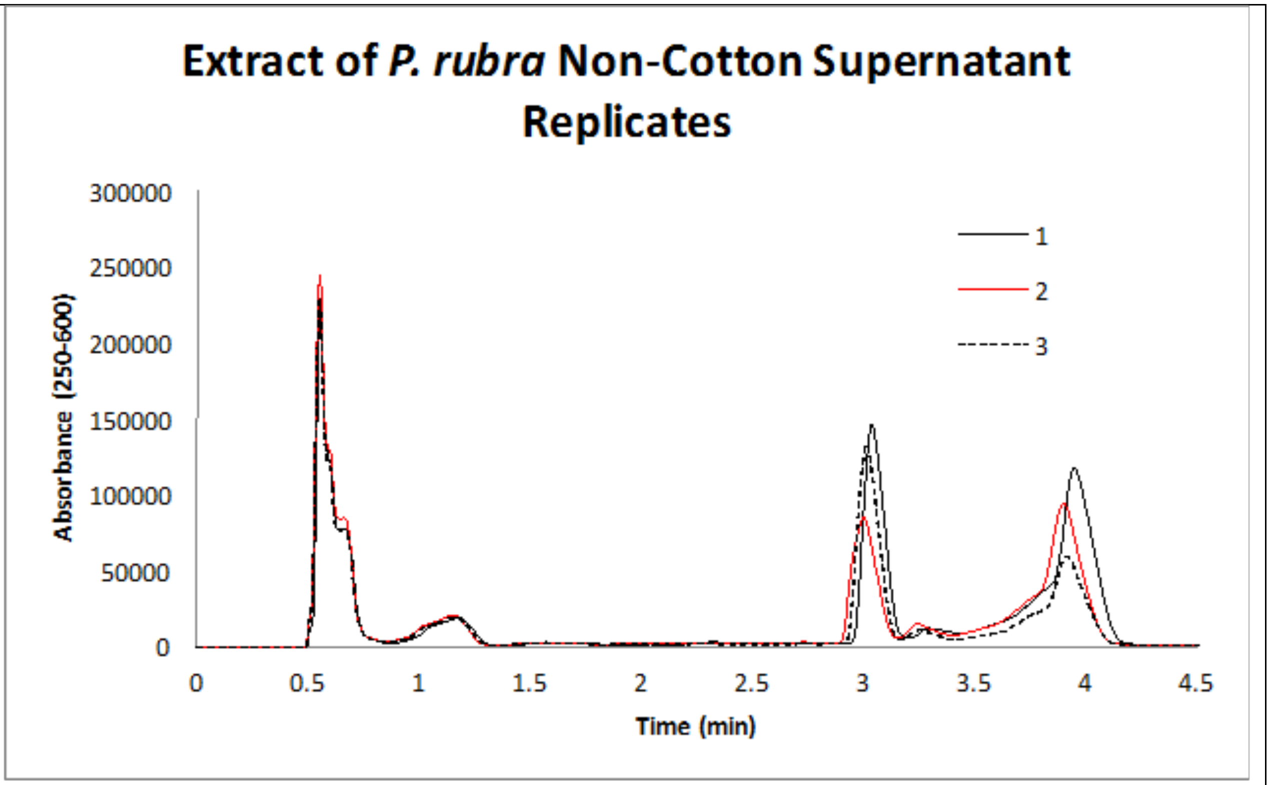

Figure S14: UPLC-PDA Traces of ethyl acetate extracts from biological replicates of planktonic cultures of *P. rubra* DSM-6842.

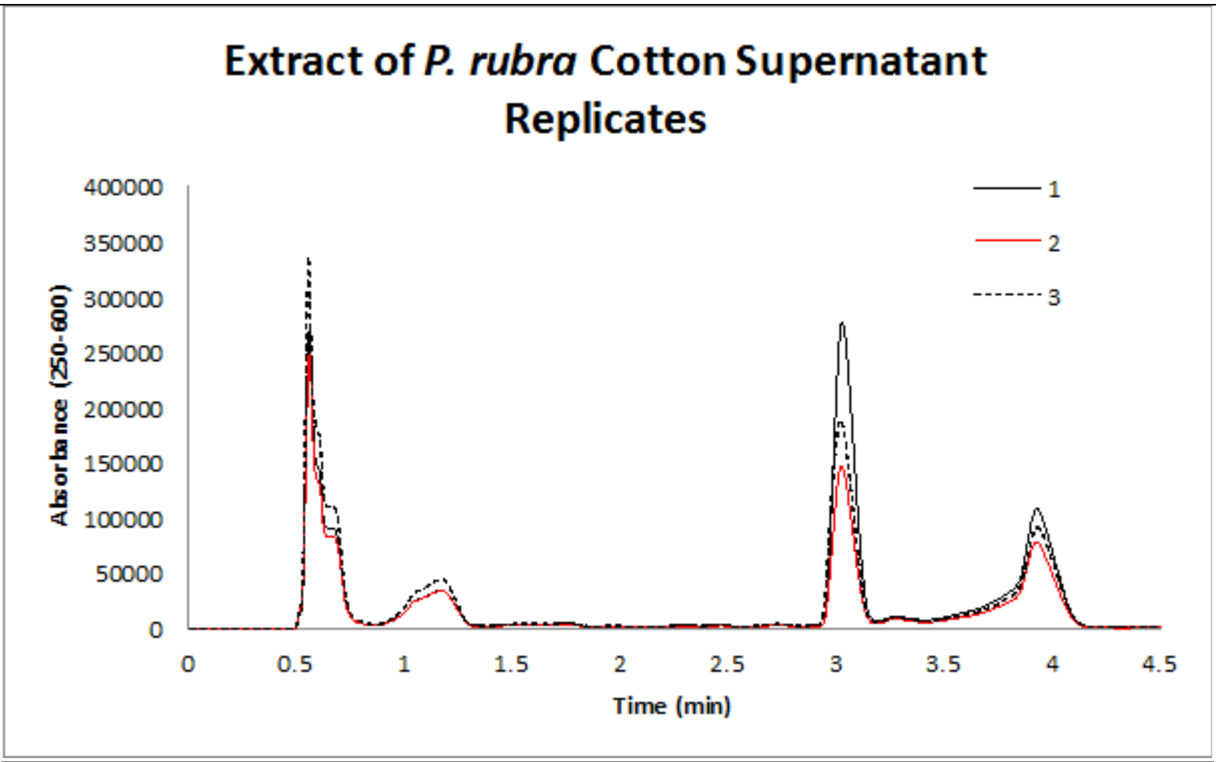

Figure S15: UPLC-PDA Traces of ethyl acetate extracts from biological replicates of cotton containing cultures of *P. rubra* DSM-6842.

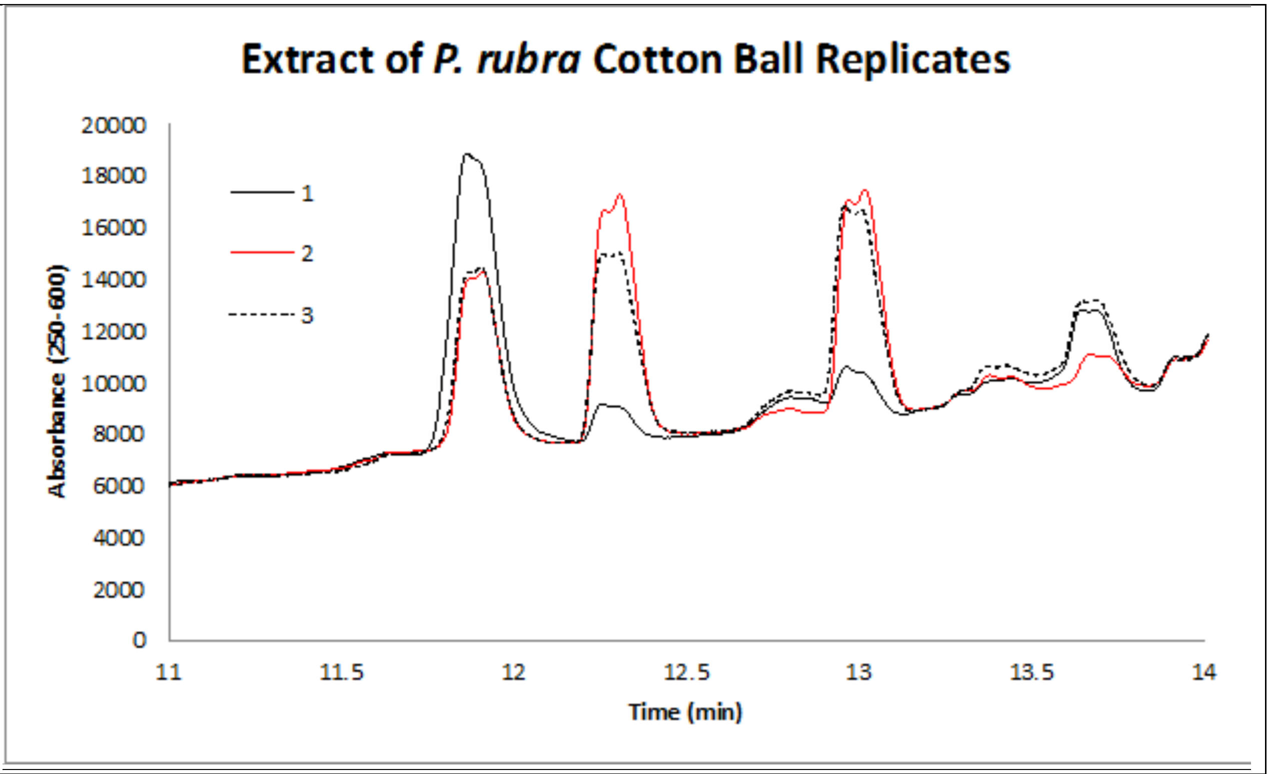

Figure S16: UPLC-PDA Traces of methanol extracts from biological replicates of cotton containing cultures of *P. rubra* DSM-6842.

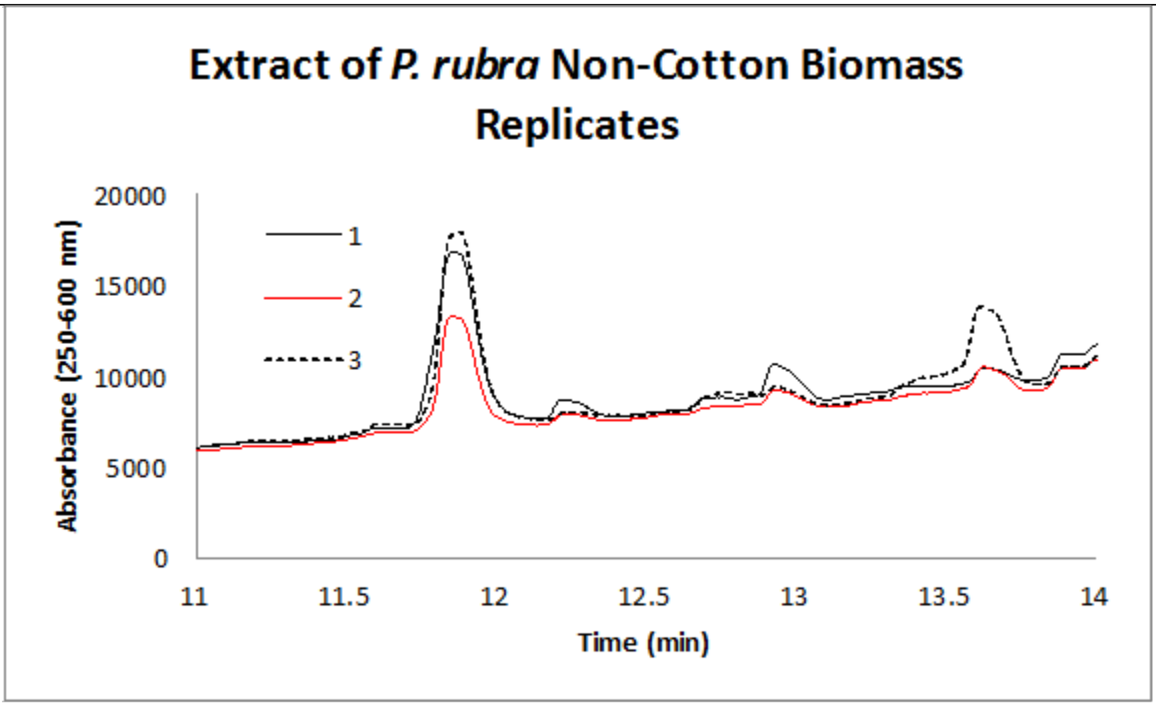

Figure S17: UPLC-PDA Trace of methanol extracts from biological replicates of planktonic cultures of *P. rubra* DSM-6842.

Table S2: Retention Times and Masses of known compounds based on dereplication using Marinlit [2]

***Pseudoalteromonas luteoviolacea* 2ta16**				
Compound	Calculated (M+H)	Observed *m/z*	Observed Adducts	Retention Time (Minutes)
Violacein	344.10	344.07	(M+Na)	16.49
Thiomarinol A	641.80	641.37	(M+Na)	19.02
***P. piscicida* JCM20779**				
Compound	Calculated (M-H)	observed *m/z*	observed adducts	Retention Time (Minutes)
Alterochromide A/A’	764.37	764.29	$(M\text{-}H+CH_2O_2)$ 810.28	11.55/11.65
Bromoalterochromide A/A’	842.28/844.28	842.15/844.09	$(M\text{-}H+CH_2O_2)$ 888.05/890.00	12.82/13.08
Alterochromide A’’	750.36	750.56	$(M\text{-}H+CH_2O_2)$ 796.30	10.94

Bromoalterochromide A''	828.27/830.27	828.10/830.09	(M-H+CH_2O_2) 874.15/876.03	12.28
Alterochromide B/B'	790.39	790.28	(M-H+CH_2O_2) 836.27	12.55/12.69
Bromoalterochromide B/B'	868.30/870.30	868.15/870.14	(M-H+CH_2O_2) 914.05/916.08	13.77/13.87
Alterochromide B''	776.37	776.24	(M-H+CH_2O_2) 822.22	12.00
Bromoalterochromide B''	854.28/856.28	854.03/856.24	(M-H+CH_2O_2) 900.12/902.27	13.26

***P. rubra* DSM-6842**

Compound	Predicted (M+H)	Observed *m/z*	Observed Adducts	Retention Time (Minutes)
Cycloprodigiosin	322.19	322.08	n/a	12.8
4''-(*n*-butyl)prodigiosin	310.14	310.04	n/a	12.8
Prodigiosin	324.21	324.06	(2M+2H+Cl^-) 683.29	13.69
4''-(*n*-hexyl)prodigiosin	338.22	338.09	n/a	14.35
4''-(*n*-heptyl)prodigiosin	352.24	352.16	n/a	14.87

Table S3: List of Other Secondary Metabolites Known to be Produced by *Pseudoalteromonas rubra, Pseudoalteromonas piscicida or Pseudoalteromonas luteoviolacea* but not detected in these experiments based on data from Marinlit [2]

P. rubra			
Compound	Molecular Mass	Reference	Notes:
2-(*p*-hydroxybenzyl)prodigiosin	429.24	DOI: 10.1021/np800493p	Strain not specified in this paper.
Pseudoalteromone A	320.38	DOI: 10.1007/s00253-014-5530-0	Described in strain QD1-2
alkyl-quinolinones	215.13, 243.16, 271.19	DOI: 10.3390/md14070129	Described in strain DSM-6842, as well as *P. piscicida* A1-J11
P. piscicida			
Norharman	168.199	DOI: 10.1042/BA20050176	Described in strain NJ6-3-1
Alteramide A	496.26	DOI: 10.3390/md14070129	Described in strain OT59
P. luteoviolacea			
Pentabromopseudilin	548.62	DOI: 10.1038/nchembio.1564	Described in strain I-L-33 and 2ta16

Various Bromophenol/bromopyrroles and their dimers	497.71, 599.53, 627.56, 525.74, 249.86, 327.77, 300.77, 378.68	DOI: 10.1038/nchembio.1564	Described in strain I-L-33 and 2ta16
Cyclo-(L-prolyl-L-glycine)	154.14	DOI: 10.1080/10575630008043781	Strain not Described
Cyclo-(L-phenylalanyl-4*R*-hydroxy-L-proline)	260.24	DOI: 10.1080/10575630008043781	Strain not Described
2,4-dibromo-6chlorophenol	283.82	DOI: 10.1080/10575630008043781	Strain not Described
Indolmycin	257.06	DOI: 10.1021/np100151y	Described in strain NCIMB 2035
4-hydroxy benzaldehyde	122.04	DOI: 10.3390/md14070129	Described in strain I-L-33
n-propyl-3-hydroxybenzoate	180.08	DOI: 10.3390/md14070129	Described in strain I-L-33
Thiomarinols B,C,D,E,F, G	672.80, 624.81, 654.83, 668.86, 638.79, 624.81	DOI: 10.3390/md14070129	Described in strain SANK 73390
Xenorhabdins,	338.15, 366.18, 336.13, 392.2, 420.23	DOI: 10.3390/md14070129	Described in strain SANK 73390

Table S4: Statistical analysis of metabolite profiles*

P. luteoviolacea			
Compound	Average Peak Area in planktonic culture (AU). [Standard Deviation]	Average peak area in cotton containing culture (AU). [Standard Deviation]	P Value (Paired Student's T test)
Violacein	0 (Not detected)	99068.27 [20543.4]	0.014
Thiomarinol A	0 (Not detected)	567851.50 [24149.8]	$6.0X10^{-4}$
P. piscicida			
Compound	Average ratio of Brominated analogue to non-brominated analogue in planktonic culture [Standard Deviation]	Average ratio of Brominated analogue to non-brominated analogue in cotton containing culture [Standard Deviation]	P Value (Paired Student's T test)
Bromoalterochromide A/A':Alterochromide A/A'	3.55 [0.276]	9.39 [1.415]	0.024

Bromoalterochromide B/B':Alterochromide B/B'	0.39 [0.148]	4.025 [1.159]	0.028

P. rubra

Compound	Average Peak Area in planktonic culture (AU). [Standard Deviation]	Average peak area in cotton containing culture (AU). [Standard Deviation]	P Value (Paired Student's T test)
Unknown compound at 12.29 minutes RT	622.6 [287.8]	9155.6 [5161.7]	0.15125
Unknown compound at 12.96 minutes RT	1586.3 [273.6]	8869.6 [5836]	0.23385
Cycloprodigiosin	10695.33 [8628.3]	25597.67 [11485.98]	0.162958
Prodigiosin	87475 [53979.75]	96010.33 [32157.31]	0.831869
4''-(*n*-hexyl) prodigiosin	12503.0 [9168.827]	14142.67 [5693.175]	0.810407
4''-(*n*-heptyl) prodigiosin	3012.333 [2300.602]	3956.333 [1580.594]	0.566131

*Analysis was performed using the Analysis ToolPak module of Microsoft Excel based on the following formula for *t* statistic:

$$t = \frac{\bar{x} - \bar{y} - \Delta_0}{\sqrt{\frac{S_1^2}{m} + \frac{S_2^2}{n}}}$$

1 Blin, K. *et al.* (2017) antiSMASH 4 . 0 — improvements in chemistry prediction and gene cluster boundary identification. *Nucleic Acids Res.* **45**, 36–41

2 Royal Society of Chemistry (2018) , MarinLit - A database of the marine natural products literature. [Online]. Available: http://pubs.rsc.org/marinlit/. [Accessed: 15-May-2018]
